# Mutations in *PSEN1* predispose inflammation in an astrocyte model of familial Alzheimer’s disease through disrupted regulated intramembrane proteolysis

**DOI:** 10.1186/s13024-025-00864-7

**Published:** 2025-06-20

**Authors:** Oliver J. Ziff, Gustavo Morrone Parfitt, Sarah Jolly, Jackie M. Casey, Lucy Granat, Satinder Samra, Núria Setó-Salvia, Argyro Alatza, Leela Phadke, Benjamin Galet, Philippe Ravassard, Marie-Claude Potier, John Hardy, Dervis A. Salih, Paul Whiting, Fiona Ducotterd, Rickie Patani, Selina Wray, Charles Arber

**Affiliations:** 1 Department of Neuromuscular Diseases, UCL Queen Square Institute of Neurology, University College London, London, UK; 2 Department of Neurodegenerative Disease, UCL Queen Square Institute of Neurology, London, UK; 3 Alzheimer’s Research UK UCL Drug Discovery Institute, University College London, London, UK; 4 Department of Clinical and Movement Neuroscience, Reta Lila Weston Institute, UCL Queen Square Institute of Neurology, London, UK; 5 Sorbonne Université, Institut du Cerveau - Paris Brain Institute - ICM, Inserm, CNRS, APHP, Hôpital de la Pitié Salpêtrière, Paris, France; 6 UK Dementia Research Institute at UCL, London, UK; 7 Human Stem Cells and Neurodegeneration Laboratory, The Francis Crick Institute, London, UK

**Keywords:** iPSC, Astrocyte, PSEN1, Alzheimer’s disease, Inflammation, Regulated intramembrane proteolysis

## Abstract

**Background:**

Mutations in *PSEN1* cause familial Alzheimer’s disease with almost complete penetrance. Age at onset is highly variable between different *PSEN1* mutations and even within families with the same mutation. Current research into late onset Alzheimer’s disease implicates inflammation in both disease onset and progression. PSEN1 is the catalytic subunit of γ-secretase, responsible for regulated intramembrane proteolysis of numerous substrates that include cytokine receptors. For this reason, we tested the hypothesis that mutations in *PSEN1* impact inflammatory responses in astrocytes, thereby contributing to disease progression.

**Methods:**

We developed patient-derived models of iPSC-astrocytes, representing three lines harbouring *PSEN1* mutations and six control lines (including two isogenic controls). Transcriptomic and biochemical assays were used to investigate differential inflammatory responses to TNFα, IL1α and C1Q.

**Results:**

We show that PSEN1 is upregulated in response to inflammatory stimuli, and this upregulation is disrupted by pathological *PSEN1* mutations. Using transcriptomic analyses, we demonstrate that *PSEN1* mutant astrocytes have an augmented inflammatory profile in their basal state, concomitant with gene expression signatures revealing dysregulated intramembrane proteolysis and JAK-STAT signalling. Detailed investigation of the JAK-STAT2 signalling pathway showed reduced cell surface expression of IFNAR2, lower STAT2 phosphorylation cascades and delayed NFκB nuclear localisation in *PSEN1* mutant astrocytes in response to inflammatory stimuli, thereby implicating the notion of altered cytokine signalling cascades. Finally, we use small molecule modulators of γ-secretase to confirm a role for PSEN1/γ-secretase in regulating the astrocytic response to inflammatory stimuli.

**Conclusions:**

Together, these data suggest that mutations in *PSEN1* enhance cytokine signalling via impaired regulated intramembrane proteolysis, thereby predisposing astrocytic inflammatory profiles. These findings support a two-hit contribution of *PSEN1* mutations to fAD pathogenesis, not only impacting APP and Aβ processing but also altering the cellular response to inflammation.

**Supplementary Information:**

The online version contains supplementary material available at 10.1186/s13024-025-00864-7.

## Background

Together with amyloid plaques and tau tangles, neuroinflammation represents one of the key pathological hallmarks of Alzheimer’s disease (AD). Both astrocytes and microglia proliferate and cluster around amyloid plaques [1] and alter their inflammation-associated transcriptomic signatures [2, 3]. It remains unclear whether inflammation is protective or harmful, however, evidence suggests that the innate immune responses may link the preclinical amyloid phase of AD to the clinical tau phase of AD [4, 5]. Recent data support a central role for astrocyte dysfunction early in AD pathogenesis, for example, via an association of astrocyte-enriched SNPs with early amyloid pathology [6], as well as changes observed to both MOA-B PET tracer positivity [7] and plasma levels of GFAP [8] prior to amyloid plaque formation. The astrocyte response to inflammatory factors released by microglia has been shown to have a central role in mediating neurodegeneration [9], although, it has been suggested that astrocyte responses may play a crucial protective role in AD [10].

Familial Alzheimer’s disease (fAD) is a rare, inherited, autosomal dominant form of AD caused by mutations in *PSEN1*, *PSEN2* and *APP* [11–13]. *PSEN1/2* encode the catalytic subunit of γ-secretase, which cleaves APP to generate Aβ, the major component of amyloid plaques [14]. Mutations in *PSEN1* destabilise the enzyme-substrate complex, thereby releasing longer forms of Aβ prior to complete processing [15–17]. These longer forms, such as Aβ42 and Aβ43, are aggregation prone and are found in plaque cores. One hypothesis linking fAD and late onset AD (LOAD) states that fAD is associated with an increased production of plaque-forming Aβ peptides, whereas LOAD is associated with decreased clearance of Aβ; this is supported by in vivo kinetic data [18, 19].

Evidence from human iPSC-derived neuronal models (which can be considered foetal-like in their maturation status) and young mouse knock-in models suggest that mutations in *PSEN1* lead to a constitutive alteration in Aβ production, i.e. from birth [20, 21]. Despite this observation, the clinical onset of fAD affects individuals in their third to seventh decades of life [22, 23], and shows considerable heterogeneity in disease onset, progression and clinical presentation, even within families with the same mutation. This suggests that, even in fAD, additional genetic or environmental factors contribute to disease onset and progression [22].

In addition to APP, γ-secretase cleaves more than 150 known substrates [24, 25], including the key inflammatory cytokine receptors IL1R [26] and TNFR1 [27]. Cell signalling can be controlled by γ-secretase-mediated cleavage, such that active signalling stubs of receptors can be cleaved for degradation (cessation of signal) or translocation (potentiation of signal) [28]. Thereby, it has been suggested that γ-secretase-mediated cleavage can modulate the inflammatory response. Examples of this include the cleavage of LRP1 and SIRPa that have been shown reduce or enhance inflammation respectively [29, 30]. More recently, a study of γ-secretase inhibition/deficiency in iPSC-derived microglia and model mice elegantly demonstrated that γ-secretase regulates key microglial genes and orchestrates cell state transitions in vivo [31]. In contrast, little is known about the effect of fAD mutations in *PSEN1* on cytokine responses and cell state transitions.

Here, we hypothesised that *PSEN1* mutations not only increase the production of aggregation-prone species of Aβ, but also alter the cellular responses to inflammatory cytokines through impaired cytokine receptor cleavage. We therefore established a patient-derived iPSC-astrocyte model to investigate the effect of *PSEN1* mutations on glial responses to inflammatory stimuli.

## Methods

### Cell culture and treatments

Patient-derived iPSCs were established under Joint Research Ethics Committee approval (09/H0716/64). All iPSC lines used in this study have been characterised and described previously, with the exception of the isogenic corrected R278 line following the protocol described previously [32] using the CRISPR/Cas9 technology [33]. Briefly, iPSC cultured in mTeSR-Plus medium were dissociated as single cells with Accutase (Stem Cell Technologies). One million cells were then transfected (4D Nucleofector system, Core Unit AAF-1002B and X unit AAF-1002; Lonza, Switzerland) with a ribonucleoprotein (RNP) complex and 500 pmol of ssODN (single stranded oligodeoxynucleotide) matrix for homologous recombination (TCTCAAATACTTACAGGAGTAAATGAGAGCTGGAAAAAGCGTTTCATTTCTCTCCTGTGCAGTTTCAACCAGCATACGAAGTGGACCTTTCGGACACAAA). The RNP complex was prepared with equimolar concentrations of crRNA (TATGCTGGTTGAAACAGCTC) and tracrRNA-ATT0550 (225 pmol of each RNA), with 120 pmol of Cas9 protein (Integrated DNA Technologies (IDT), Iowa, USA). After transfection, all cells were plated and the day after ATT0550-positive cells were sorted by FACS (MoFlo Astrios, Beckman Coulter; ICV-Cytometrie, ICM, Paris, France) and plated at low concentrations on petri dishes coated with Laminin521 (Stem Cell Technologies) in mTeSR-Plus medium supplemented with Clone R (Stem Cell Technologies). Seven to 10 days later, individual iPSC clones were picked and expanded in 96-well plates. Each clone was duplicated for either DNA analysis or freezing. Between 100 and 200 clones were picked up. Genetically engineered clones were screened after DNA extraction by PCR amplification of the targeted genomic region and sequencing using the following primers: FWD TCCTCCCTACCACCCATTTAC and REV GGAGTTCCAGGAATGCTGTG (Fig. S1). Corrected clones were then amplified and characterized at the molecular level. Potential off-target sites with one, two or three mismatches were screened by Sanger sequencing (Eurofins Genomics). No off-target mutations were found in the selected clones. Corrected clones were tested for recurrent genomic abnormalities by the ICS-digital™ PSC test (Stem Genomics, Montpellier, France).

Details of iPSC lines are described in Table 1. iPSCs were cultured in Essential 8 media on Geltrex substrate and passaged using EDTA mechanical passaging. All components were purchased from ThermoFisher and all recombinant proteins were Peprotech, unless specified. Sanger sequencing was performed with SourceBio. Low coverage whole genome sequencing was performed in collaboration with UCL Genomics. Reads were divided into 1000 kb bins and the QDNASeq package was employed that included smoothing and control for mapability [34].

**Table 1:** Cell lines used in this study

iPSC line	Previously described	Male/Female	Age at onset
Control 1	Ctrl1 (20)	Male	-
Control 2	ND41886 (20)	Male	-
Control 3	RBi001 (20)	Male	-
Control 4	SIGi1001 (20)	Female	-
Isogenic control to PSEN1 int4del	(35)	Female	-
Isogenic control to PSEN1 R278I	This study	Male	-
PSEN1 int4del	(20)	Female	47
PSEN1 Y115H	(20)	Male	34
PSEN1 R278I	(20)	Male	58

Human foetal astrocytes were purchased ScienCell Research Laboratories (catalogue #1800) and maintained and passaged in an identical manner to the iPSC-derived astrocytes.

To generate iPSC-derived cortical neurons, we followed established protocols [20, 35–38]. In brief, neural induction was performed using SB431542 (10µM, Tocris) and dorsomorphin (1µM, Tocris) in N2B27 media. N2B27 media is composed of 50% DMEM-F12, 50% Neurobasal with the addition of 0.5X N2 supplement, 0.5X B27 supplement, 0.5X L-glutamine, 0.5X NEAA, 0.5X pen/strep (25U), 1:1000 b-mercaptoethanol, and insulin at 25U. 100 DIV was used as the final time-point.

For the generation of iPSC-astrocytes we followed established protocols [39–41]. Glial precursor cells (GPCs) were enriched from neuronal cultures at 80 DIV by regular passaging using EDTA and the addition of FGF2 (10ng/ml, Peprotech 17850673) to the N2B27 media. A gliogenic switch occurred over the course of the FGF2 propagation phase. To generate mature astrocytes, GPCs of at least 150 DIV were subjected to two weeks maturation with 10ng/ml BMP4 (Thermo PHC9534) and 10ng/ml LIF (Sigma, L5283) in N2B27 media.

iPSC-microglia were generated using established protocols [41, 42]. Briefly, haematopoiesis was induced in iPSC-derived embryoid bodies via treatment with 50ng/ml BMP4, 50ng/ml VEGF and 20ng/ml SCF. Myeloid patterning was performed using MCSF (100ng/ml) and IL3 (25ng/ml) in XVIVO media (Lonza). Microglia progenitors were harvested from the embryoid bodies supernatants and matured in media containing MCSF (25ng/ml), IL34 (100ng/ml) and TGFb (5ng/ml), with a final two-day treatment in CX3CL1 (100ng/ml) and CD200 (100ng/ml).

Conditioned media was collected after 48 h and spun at 2,000G to remove cell debris, prior to using for Western blotting, ELISA, cytokine arrays or LDH assays.

Cell treatments included TIC, where TNFα (33ng/ml, Peprotech), IL1α (3ng/ml Peprotech) and C1Q (400ng/ml, Merck) were used in combination for 24 h. γ-secretase modulation was performed with E2012 (GSM, 10µM, MedChemExpress). γ-secretase inhibition was performed using DAPT (GSI, 10µM, Tocris). For treatments with GSI/GSM in combination with TIC a 1-hour pre-treatment with GSI/GSM was performed prior to the addition of TIC.

### Immunocytochemistry and high content imaging

Cells were fixed using 4% paraformaldehyde for 15 min. Three washes were performed in PBS with 0.3% triton-x-100 (PBSTx). Blocking was done for 20 min using 3% bovine serum albumin in PBSTx. Primary antibodies (Table 2) were incubated in blocking solution overnight at 4 °C. Secondary antibodies (AlexaFluor) were incubated for 1 h in blocking solution after 3 washes in PBSTx. DAPI was used as a counterstain with final washes. Imaging was done using the high content imaging system Opera Phenix (Perkin Elmer) and analysis was done on the Columbus software. No post-hoc image processing was performed.

### Flow cytometry analysis

Astrocytes were plated in 6-well plates coated with Geltrex and recovered for 3 days before the experiments. After being treated with TIC for 24 h, the astrocytes were dissociated with Accutase for 5 min, washed, and incubated with 3% BSA in PBS and Human TruStain FcX™. Cells were then incubated for 1 h with CD44-FITC, IFNAR2-APC (Table 2) and Zombie Violet™ viability dye. After washing three times in PBS, the cells were fixed in 2% PFA and analysed in a BD FACSymphony™ A5 SE cytometer. Data was analysed in FlowJo_v10.

### Western blotting

Cells were lysed in RIPA buffer containing phosphatase and protease inhibitors (Roche) and spun to remove insoluble debris. Protein content was quantified using the BioRad BCA assay. Samples were denatured in LDS with DTT and loaded onto 4–12% precast polyacrylamine gels. Gels were transferred to nitrocellulose membranes and then blocked in PBS with 0.1% Tween20 (PBSTw) with 3% bovine serum albumin. Primary antibodies (Table 2) were incubated in blocking solution overnight, before 3 washes in PBSTw and secondary antibody incubation (Licor) for 1 h. Membranes were imaged using a Licor Odyssey fluorescent imaging system. Quantification was done on the ImageStudio software.

**Table 2:** Antibodies for ICC and Western blotting

Antigen	Species	Company (catalogue number)	RRID
SOX9	rabbit	Abcam ab185966	RRID: AB_2728660
GFAP	mouse	Sigma G6171	RRID: AB_1840893
ALDH1L1	rabbit	Abcam ab87117	RRID: AB_10712968
Phalloidin	-	SantaCruz Sc-363797	
pMAPK	rabbit	CST 9101	RRID: AB_331646
MAPK	rabbit	CST 9102	RRID: AB_330744
pSTAT2	rabbit	CST 88410	RRID: AB_2800123
STAT2	rabbit	Abcam Ab32367	RRID: AB_778098
pSTAT3	rabbit	CST 9145	RRID: AB_2491009
STAT3	rabbit	CST 4904	RRID: AB_331269
pNFκB	rabbit	CST 3033	RRID: AB_331284
NFκB	rabbit	CST 8242	RRID: AB_10859369
APP	rabbit	Thermo A8717	RRID: AB_258409
PSEN1	rat	Millipore MAB1563	RRID: B_11215630
PSEN2	rabbit	Cell Signalling Technology 9979	RRID: AB_10829910
Actin	mouse	Sigma A1978	RRID: AB_476692
GAPDH	mouse	Ambion AM4300	RRID: AB_437392
sAPPβ	rabbit	IBL JP18957	RRID: AB_1630824
sAPP total	mouse	(22c11) Millipore MAB348	RRID: AB_94882
CD44-FITC	mouse	Biolegend 338803	RRID: AB_1501204
IFNAR2-APC	human	Miltenyi Biotec 130-099-560	RRID: AB_2652223

### Quantitative PCR

For qPCR, cells were lysed in Trizol and RNA was isolated following the manufacturer’s protocols. cDNA was synthesised from 1 to 2 µg of RNA using Superscript IV with random hexamer primers and RNAse OUT. qPCR was run using Power SYBR green on an Agilent Aria MX machine with annealing at 60 °C. Samples were analysed relative to the housekeeping gene *RPL18a* and relative expression was quantified using the ΔΔCT method. qPCR primers are listed in Table 3.

**Table 3:** Primers used for qPCR

Target	Forward	Reverse	Amplicon size
*RPL18A*	CCCACAACATGTACCGGGAA	TCTTGGAGTCGTGGAACTGC	180 bp
*ISG15*	AGATCACCCAGAAGATCG	ATGCTCAGAGGTTCGTC	160 bp
*OAS1*	AAGCCTGTCAAAGAGAGAGAGC	GGTTAGGTTTATAGCCGCCAG	180 bp
*CXCL10*	TCCTCAATTGCTTAGACATA	AGAGAGGTACTCCTTGAATG	132 bp

### RNA sequencing

Cells were lysed in Trizol and RNA was harvested using the Monarch total RNA miniprep kit (NEB).

Poly(A) + selected sense-stranded RNA sequencing libraries were prepared using the KAPA mRNA Hyper Prep (Roche), with 500 ng of total RNA as input. Libraries were sequenced on the NextSeq 2000 platform for neuronal data and NovaSeq 6000 for astrocyte data. A mean of 189 (range 97–284) million 55 bp paired-end strand-specific reads were sequenced per sample. mRNA sequencing reads were processed using the nf-core/rna-seq v3.11.2 pipeline [43]. Raw reads underwent adaptor trimming with Trim Galore, removal of ribosomal RNA with SortMeRNA, alignment to Ensembl GRCh38.99 human reference genome using splice-aware aligner, STAR v2.6.1 and BAM level quantification with Salmon to enable transcript level counting. Detailed quality control of raw and aligned reads were assessed by utilizing FastQC, RSeQC, Qualimap, dupRadar, Preseq and MultiQC tools. All libraries generated in this study had < 0.1% rRNA, < 0.3% mismatch error, and > 85% strandedness.

### Transcriptomic analysis

STAR aligned and Salmon quantified transcript abundance were summarised at the gene-level using tximport in R v4.2.0. Gene counts were normalized and transformed using the variance stabilizing transformation function in DESeq2 [44]. These transformed values were utilized in the principal component analysis (PCA) and unsupervised hierarchical clustering. We examined the astrocyte transcriptomic identities of iPSC-astrocytes using the ComplexHeatmap package based on the expression of canonical neuronal and glial cell type markers.

Differential gene expression analysis was fitted using DESeq2 and normalised using the mean of ratios. Results of *PSEN1* mutant iPSC-astrocytes were generated by comparing the *PSEN1* mutant versus non mutant control samples using the Wald test. To examine the effect of TIC on gene expression we compared the TIC treated versus untreated samples. Results for *PSEN1* mutant astrocytes were correlated with TIC treated astrocytes by matching the Wald test statistic for each gene followed by Pearson correlation. In all analyses, genes were considered differentially expressed at FDR < 0.05. Significantly up- and down-regulated differentially expressed genes were used as input to functional over-representation analyses to identify enriched pathways using g: Profiler2. g: Profiler2 searches the following data sources: Gene Ontology (GO; molecular functions, biological processes and cellular components), KEGG, REAC, WikiPathways, CORUM and Human Phenotype Ontology. g: Profiler2 reports the hypergeometric test *p*-value with an adjustment for multiple testing using the Bonferroni correction. Over-represented function categories are plotted in bar charts, where the top significant terms were manually curated by removing redundant terms. The decoupleR package was used to estimate PROGENy signalling pathway activities and DoRothEA TF regulon activities inferred from gene expression changes [45]. PROGENy and DoRothEA weights are based on perturbation experiments that are not specific to motor neurons. Their signalling pathways may activate diverse downstream gene expression programmes depending on the cell type and perturbing agent utilised [46].

To assess pathway inflammatory activity, gene sets were retrieved from the MSigDB C2 package, filtering for gene sets containing inflammatory manipulations (Table S2). Single-sample gene set enrichment analysis (ssGSEA) was then applied to each gene set using the GSVA package, generating enrichment scores for each sample [47]. The resulting ssGSEA scores were visualised as a heatmap.

### Cytokine array

The Proteome Profiler Human XL Cytokine Array Kit (R&D Systems) was used, as per the manufacturer’s instructions. Conditioned media from three independent batches were pooled (160 µl x 3) to generate an average reading per genotype and per condition. As a result of this pooling, relative cytokine abundance was not compared between genotypes.

### Lactate dehydrogenase assay

Lactate dehydrogenase assays were performed on conditioned media to quantify cell death. The Abcam LDH assay kit (ab65393) was used following the manufacturers instruction and the final absorbance was read on a Tecan SPARK 10 M plate reader at 450 nm.

### ELISAs

Aβ40 and Aβ42 were quantified using the Meso Scale Discovery V-Plex Aβ peptide panel (6E10). Samples were diluted 2-fold and quantification was performed on an MSD Sector 6000.

To quantify secreted TNFR1, we used the Quantikine Human TNF RI/TNFRSF1A ELISA from R&D Systems (DRT100), following the manufacturer’s protocols. Absorbance was measured on a Tecan SPARK 10 M plate reader.

### Glutamate uptake assay

Methods were adapted from published work [48, 49]. 15,000 astrocytes were seeded per well of a 96 well plate. Prior to assay, cells were cultures in HBSS without magnesium and calcium for 30 min. Astrocytes were then cultured in HBSS with calcium and magnesium containing 100µM Glutamate for 5 h. Conditioned media was then harvested and glutamate was quantified using a colorimetric assay (Abcam ab83389) using a Tecan Spark plate reader.

### Statistics

Data were collected and analysed using Microsoft Excel and GraphPad Prism. Normality was tested using D’Agostino and Pearson test. Comparisons between two groups (qPCR and Western blot data) were performed using two tailed t-tests. FACs data was analysed using two-way ANOVA with Tukey’s multiple comparisons test. Data is shown as *p* < 0.05 = *, *p* < 0.01 = **, *p* < 0.001 = *** and *p* < 0.0001 = ****.

Outliers were identified if they had a z-score of above 3 or below − 3. Z scores were calculated as (sample - mean)/standard deviation, for normally distributed data.

## Results

### Development of an iPSC-derived astrocyte model of fAD

To test the hypothesis that mutations in *PSEN1* alter glial inflammatory responses, we generated patient iPSC-derived astrocyte models of fAD following an established serum-free protocol [39, 40]. We focused our investigations on astrocytes, as these cells represent an immune competent cell type that expresses high levels of both APP and PSEN1 (Fig S2). We analysed three fAD patient-derived lines (with the int4del, Y115H and R278I mutations in *PSEN1*) with six healthy control lines (two of which were isogenic corrected lines– Table 1). As an established inflammatory cue, we use TNFα, IL1α and C1Q [9], hereafter referred to as TIC and performed deep RNA sequencing using poly(A) strand specific sequencing on paired samples (see Methods).

**Fig. 1:**
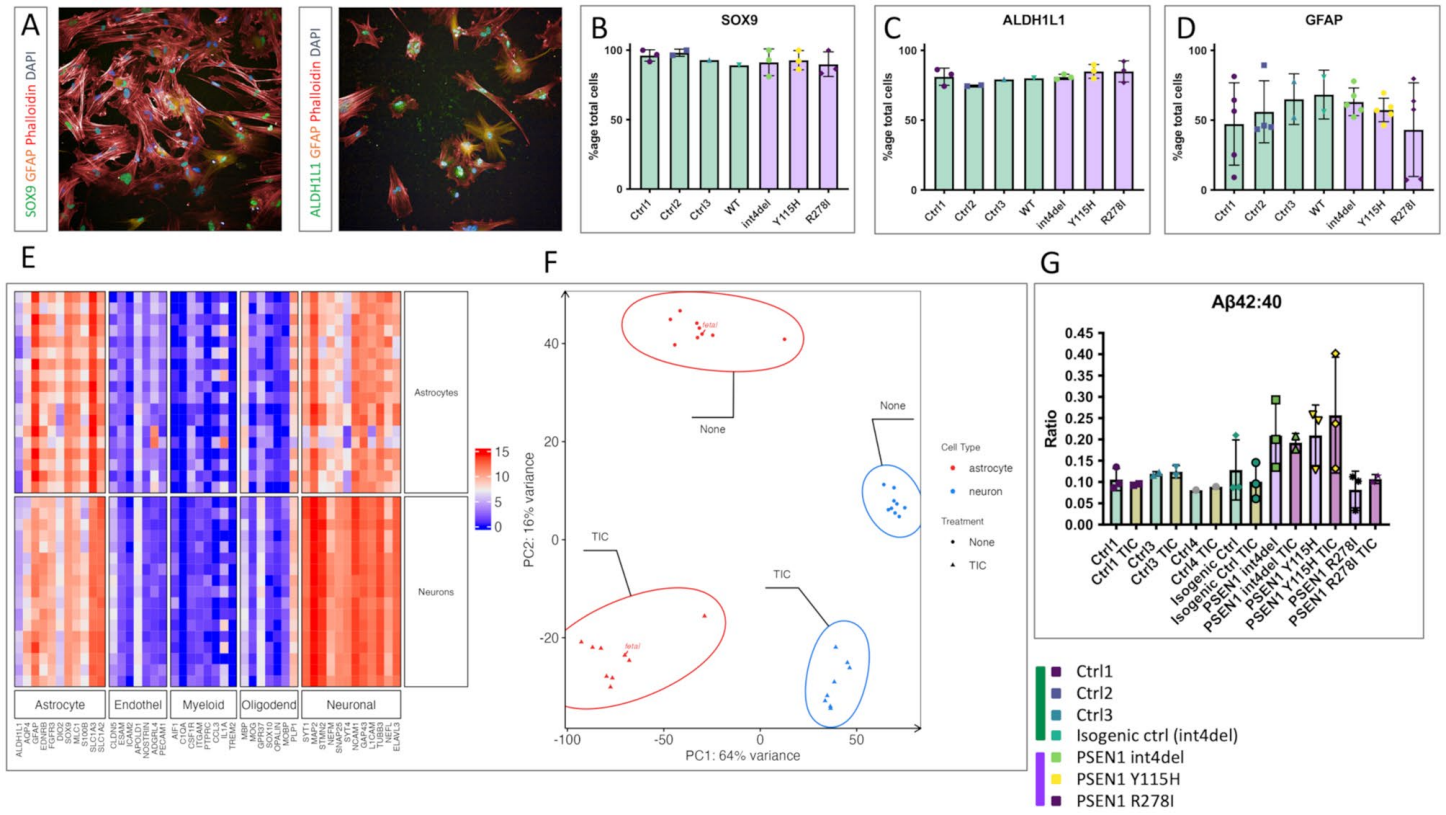
Establishment of an iPSC-derived astrocyte model of familial Alzheimer’s disease. (**A**) Immunocytochemistry of iPSC-astrocytes for markers of astrocytes (SOX9, GFAP, ALDH1L1) and counterstained with phalloidin (f-actin) and DAPI (DNA). (**B-D**) Characterisation of iPSC-astrocyte cultures using high content imaging showing the percentage of cells exhibiting positive immunostaining for astrocyte markers (data represent 3 independent experimental replicates from 4 control lines and 3 fAD lines total– see Table S1. Technical duplicates are shown for GFAP analysis). (**E**) Transcriptomic characterisation of iPSC-astrocyte cultures in comparison to iPSC-neuronal cultures, showing a relative enrichment of astrocyte markers (data represent 3 independent experimental replicates from 5 control lines and 3 fAD lines total– see Table S1). (**F**) Principal component analysis of transcriptomic data from iPSC neurons, iPSC astrocytes and primary foetal astrocytes, either untreated (none) or stimulated with TNFα, IL1α and C1Q (TIC) for 24 h. (**G**) ELISA-based quantification of Aβ42 and Aβ40 in iPSC-astrocyte cultures. The ratio of Aβ42:40 is shown, a well-established biomarker of fAD (data represent 3 independent experimental replicates from 4 control lines and 3 fAD lines total– see Table S1)

We first confirmed the identity of our iPSC-derived astrocyte cultures. Using immunocytochemistry we found homogeneous expression of the astrocyte markers SOX9 and ALDH1L1 (Fig. 1A-C). Ubiquitous SOX9 positivity enables confidence in the cellular identity, given its role in astrocytic specification [50]. We observed a lower proportion of GFAP immunoreactivity, potentially reflective of lower inflammatory states in these serum-free cultures (Fig. 1D) [51]. Transcriptomic analysis confirmed enrichment of astrocyte marker genes in our iPSC-derived astrocyte cultures compared with iPSC-derived neurons, including commonly used *S100B*, *SLC1A3* (*EAAT1*/*GLAST*) and *SLC1A2* (*EAAT2*/*GLT1*) (Fig. 1E). Principal component analysis revealed separation based on cell type along PC1 and we found that human foetal astrocytes clustered together with our iPSC-astrocytes (Fig. 1F). We next examined responses to TIC treatment and confirmed TIC treated samples separated from their untreated counterparts along PC2 (Fig. 1F). Cytokine arrays demonstrated the upregulation of inflammatory cytokines and chemokines in astrocyte conditioned media after TIC treatment (Fig S2B).

Importantly, TIC treatment altered neither the Aβ profiles of the astrocyte cultures (Fig. 1G) nor measures of cell death (Fig S2C). It should be noted that although Aβ profiles resembled previously described ratios in iPSC-neurons [20], Aβ levels were considerably more variable in astrocyte conditioned media, perhaps reflecting lower amyloidogenic processing (lower levels of sAPPβ and APP CTFs with lower molecular weight (CTFα), Fig S2A, Fig S2D-E). Glutamate uptake assays demonstrated the functionality of the iPSC-derived astrocytes (Fig S2F), and TIC treatment reduced glutamate uptake, in line with previous studies [48, 49]. Thus, these patient-derived cultures represent a physiological model of fAD astrocytes capable of responding to inflammatory cues.

### PSEN1, PSEN2 and APP are involved in astrocytic responses to inflammatory cues

**Fig. 2:**
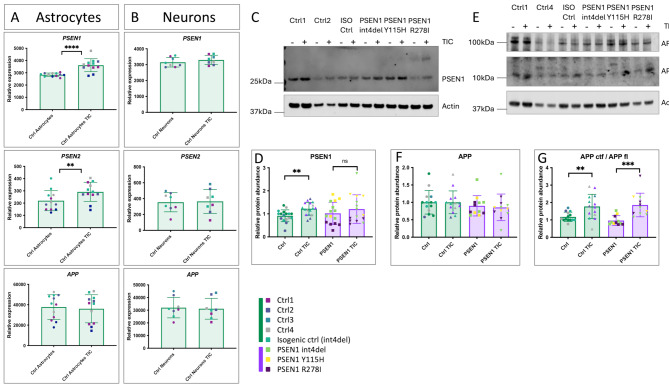
PSEN1, PSEN2 and APP are involved in astrocytic responses to inflammatory cues. *PSEN1* mutations impinge on PSEN1 upregulation. (**A**) Expression of *PSEN1*, *PSEN2* and *APP* in control astrocytes in basal conditions and after 24 h TNFα, IL1α, C1Q (TIC). Data represent 3 independent experimental replicates from 5 control lines– see Table S1). (**B**) Expression of *PSEN1*, *PSEN2* and *APP* in control neurons in basal conditions and after 24 h TNFα, IL1α, C1Q (TIC). Data represent 2 independent experimental replicates from 4 control lines– see Table S1). (**C**) Western blotting of iPSC-astrocyte lysates for PSEN1. Actin is shown as a loading control. (**D**) Quantification of PSEN1 Western blotting. Data represent up to 4 batches of astrocytes and up to 5 experimental replicates, including 5 control lines and 3 fAD lines (see Table S1). (**E**) Western blotting of iPSC-astrocyte lysates for APP. Actin is shown as a loading control. (**F-G**) Quantification of APP Western blotting. Data represent up to 4 batches of astrocytes and up to 4 experimental replicates, including 5 control lines and 3 *PSEN1* mutant lines (see Table S1). For data separated by each iPSC line, see Fig S7. Pairwise comparisons represent two tailed t-tests, where * = *p* < 0.05, ** = *p* < 0.01, *** = *p* < 0.001, and **** = *p* < 0.0001

Based on the literature demonstrating γ-secretase cleavage of cytokine receptors [26, 27], we investigated the role of PSEN1, PSEN2 and APP in the astrocytic response to inflammatory cues. In healthy control astrocytes, *PSEN1* and *PSEN2* gene expression is significantly increased in response to TIC (Fig. 2A). This contrasts with iPSC-derived neuronal cultures, which show no significant change in expression (Fig. 2B). The upregulation of PSEN1 at the protein level was confirmed by Western blotting in iPSC-astrocytes from healthy controls (Fig. 2C-D). TIC treatment in *PSEN1* mutant astrocytes led to a non-significant increase in PSEN1. Gene and protein expression of *APP* was not significantly altered by TIC in iPSC-derived astrocytes (Fig. 2A); however, we observed a significant accumulation of APP C-terminal fragments upon TIC treatment, representing the substrate for γ-secretase cleavage (Fig. 2E-G).

Together, these data suggest that fAD-associated genes are involved in glial responses to inflammation. Moreover, *PSEN1* mutations disrupt the upregulation of PSEN1 at the protein level, potentially impacting its role in inflammatory responses.

### *PSEN1* mutant astrocytes show altered membrane protease gene expression

Regulated intramembrane proteolysis is orchestrated by numerous enzymes. Prior to γ-secretase cleavage, ectodomain shedding of substrates is required to enable loading of the membrane domain into the γ-secretase complex [52]. Shedding is commonly attributed to α-secretases (ADAM10 and ADAM17) or β-secretase (BACE1).

**Fig. 3:**
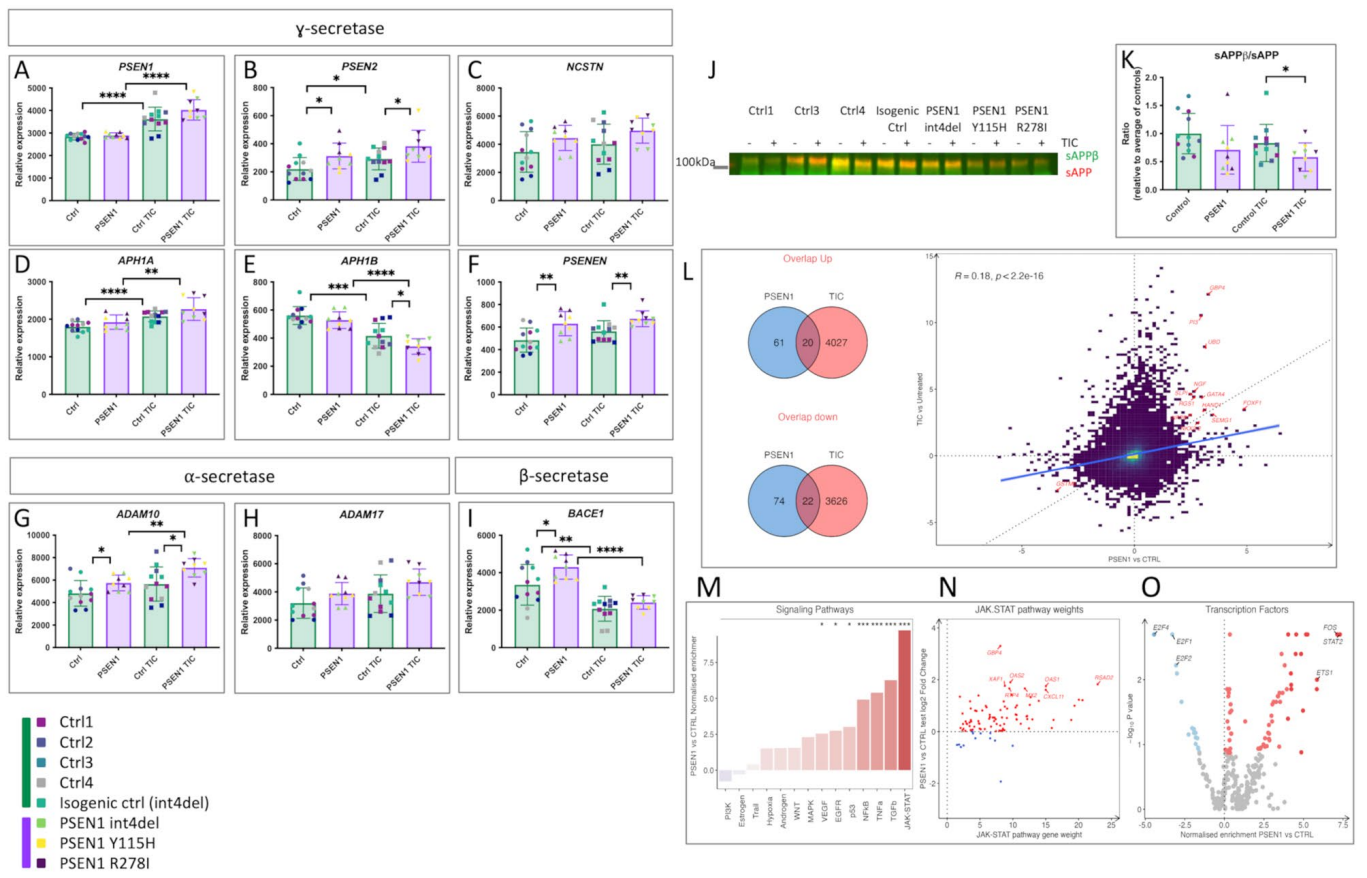
*PSEN1* mutations disrupt regulated intramembrane proteolysis and lead to inflammatory phenotypes. (**A-I**) Expression of γ-secretase, α-secretase and β-secretase components in basal and TIC-treated iPSC-astrocytes, as quantified by transcriptomic analysis. Data represents 3 experimental repeats with 5 controls and 3 *PSEN1* mutant astrocyte lines. (**J**) Western blot analysis for sAPPβ and total sAPP in conditioned media from iPSC-astrocytes, quantified in (**K**). Full blot shown in Fig S9. Data represents 3 experimental repeats with 5 controls and 3 *PSEN1* mutant astrocyte lines (see Table S1). (**L**) Correlation analysis of differentially expressed genes (DEGs) in control TIC-treated astrocytes and *PSEN1* mutant untreated astrocytes, relative to control untreated cultures. Of 175 DEGs in *PSEN1* mutant cultures, 42 are shared with TIC treated controls. (**M-O**) Regulon analysis to implicate transcription factors and cell signalling pathways driving differential gene expression in untreated *PSEN1* versus control cultures, analysed via DoRothEA. JAK-STAT2 signalling is highlighted as the most significant pathway. Pairwise comparisons represent two tailed t-tests, where * = *p* < 0.05, ** = *p* < 0.01, *** = *p* < 0.001, and **** = *p* < 0.0001

We investigated the expression of γ-secretase, β-secretase and α-secretase components in iPSC-derived astrocytes and the effect of *PSEN1* mutations and inflammatory cues (TIC). As described above, *PSEN1* was upregulated by inflammatory conditions (Fig. 2A). Mutations in *PSEN1* did not affect *PSEN1* gene expression itself (Fig. 3A). However, *PSEN2* was significantly increased in *PSEN1* mutant astrocytes compared to healthy controls (Fig. 3B), possibly reflecting a compensatory response.

In other γ-secretase components, we witnessed upregulated expression in response to TIC with varying degrees of statistical significance (Fig. 3C-F) (*APH1B* was downregulated in contrast to its alternate subunit *APH1A*). Interestingly, there was a trend to increased expression of all γ-secretase components at basal levels in *PSEN1* mutant astrocytes compared to healthy controls, mirroring the effect of TIC treatment in healthy control astrocytes. This was most significant in the upregulation of the obligate γ-secretase component *PSENEN*, a subunit for which there is no redundancy (Fig. 3F). α-secretase-associated *ADAM10* and *ADAM17* both showed trends towards upregulation in response to inflammation, and *PSEN1* mutations caused significantly upregulated *ADAM10* expression (Fig. 3G-H). Expression of the β-secretase gene *BACE1* was reduced by inflammatory stimuli, and we found a significant upregulation in basal conditions in *PSEN1* mutants compared to healthy control patient-derived astrocytes (Fig. 3I).

To further investigate altered regulated intramembrane proteolysis, we quantified sAPPβ relative to total sAPP in astrocyte conditioned media (Fig. 3J-K). We observed a non-significant reduction in relative sAPPβ levels after addition of TIC. We observed a significant reduction in β-secretase cleavage of APP in *PSEN1* mutant cells under inflammatory conditions, compared to controls, suggesting an altered balance of β-secretase versus α-secretase-mediated cleavage. We then measured the shed ectodomain of TNFR1, sTNFR1, in astrocyte conditioned media as TNFR1 is a known substrate of γ-secretase [27]. We observed a non-significant trend to increased sTNFR1 in fAD conditioned media in basal conditions (Fig S3A-B). TIC treatment had little effect on TNFR1 shedding.

Together, these data suggest that inflammatory stimuli alter the expression of genes associated with regulated intramembrane proteolysis. In untreated *PSEN1* mutant astrocytes, we observe significantly altered gene expression in a similar direction to the changes seen in response to inflammation, suggesting that inflammatory state may be affected by the presence of mutations in *PSEN1*.

### *PSEN1* mutant astrocytes display augmented inflammatory states

To further investigate the consequence of *PSEN1* mutations on astrocyte function, we performed transcriptomic analysis in untreated and TIC-treated astrocytes.

We found 272 differentially expressed genes (DEGs) in *PSEN1* mutant astrocytes versus healthy control iPSC astrocytes, with 115 upregulated and 157 downregulated DEGs (Fig S4A). Gene Set Enrichment analysis (GSEA, KEGG) showed that DEGs were overrepresented by terms such as cytokine-cytokine receptor (GAGE analysis demonstrates 132 genes with an adjusted *P* value of 1.1 × 10^− 1^) (Fig S5) [53, 54]. The TNFα pathway was also significantly upregulated (GAGE analysis demonstrates 91 genes with an adjusted *P* value of 8.5 × 10^− 2^), as was the Alzheimer’s disease term (305 genes with an adjusted *P* value of 1.4 × 10^− 1^). These data support the hypothesis that *PSEN1* mutations may impinge on regulated intramembrane proteolysis.

We found 2,968 differentially expressed genes in TIC-treated versus untreated healthy control iPSC-astrocytes, with 1746 upregulated and 1222 downregulated in TIC-treated astrocytes (Fig S4B). 3,374 genes were differentially expressed between untreated and TIC-treated astrocytes with the *PSEN1* genotype, with 1,884 upregulated and 1,490 downregulated (Fig S4C). Functional over-representation analysis (KEGG) revealed similar pathways in TIC-treated versus untreated controls for both genotypes. Upregulated pathways included cytokine-cytokine receptor interaction, TNF signalling and NFκB signalling pathways. Downregulated genes did not enrich for KEGG pathways.

When investigating the DEGs compared to control untreated cultures, we observed a significant overlap in *PSEN1* mutant cultures and TIC treated healthy controls (Fig. 3L). Of 175 DEGs between untreated controls and untreated *PSEN1* astrocytes, 42 were shared with genes upregulated by TIC in control cultures (up- and downregulated). This suggests similarities between the TIC induced inflammatory phenotype and *PSEN1* mutant astrocytes in basal conditions. Further, we performed a gene set enrichment analysis generating a transcriptional score from our lines against 14 published datasets that quantify inflammatory responses (Table S2). When ranked, *PSEN1* mutant astrocytes display an increased inflammatory score, supportive of a higher inflammatory phenotype compared to controls (Fig S4D).

To understand how signalling pathways are activated in *PSEN1* mutant astrocytes under basal conditions, we performed a Signalling Pathway RespOnsive GENes (PROGENy) analysis [45]. This revealed that the most substantial increase in pathway activity in *PSEN1* mutants was in the JAK-STAT pathway, followed by TGF, TNFα, and NFκB (Fig. 3M). Examining each gene in the JAK-STAT pathway according to its JAK-STAT weighting revealed that the genes with the strongest responsiveness in JAK-STAT activity in *PSEN1* mutant astrocytes included *RSAD2*, *OAS1*, *OAS2*, *CXCL11*, and *GBP4* (Fig. 3N). Notably, the JAK-STAT pathway was also substantially upregulated in the TIC response in healthy control astrocytes (Fig S6).

We next inferred the activities of 429 transcription factors (TFs) from their regulon expression within the DoRothEA database. This revealed that STAT2 was the transcription factor with the greatest increase in activity in basal *PSEN1* mutant astrocytes (Fig. 3O) followed by FOS and ETS1.

### JAK-STAT2 signalling is activated in *PSEN1* mutant astrocytes

**Fig. 4:**
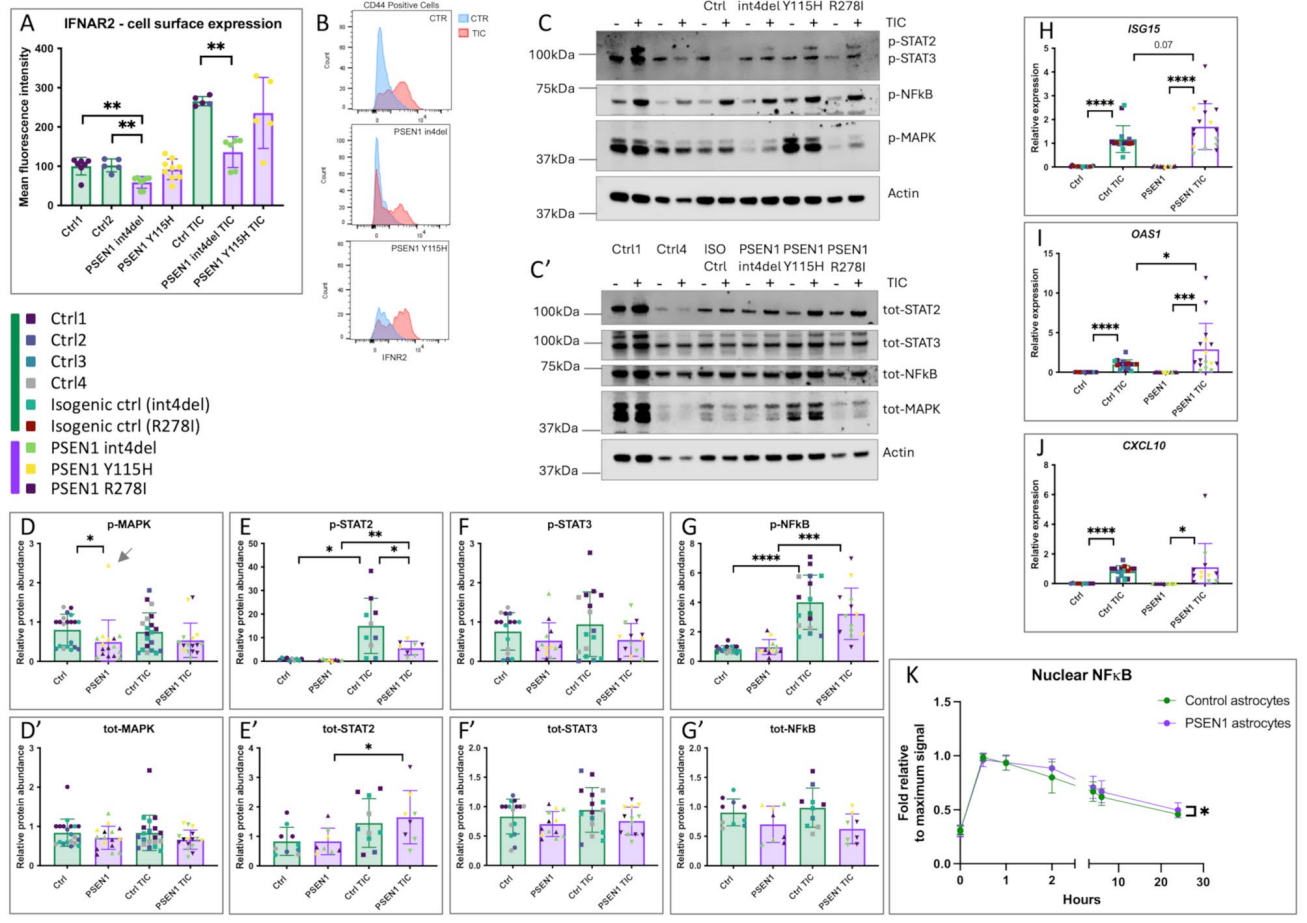
JAK-STAT signalling pathways are disrupted in *PSEN1* mutant astrocytes. (**A-B**) FACs analysis of cell surface levels of IFNAR2 in CD44-positive iPSC-astrocytes, with and without TIC treatment. Data represent 5 experimental repeats from two batches and include 2 control lines and 2 *PSEN1* mutant lines. Note that two control lines are pooled for TIC-treated datapoint. Data analysed within treatment subgroups via one-way ANOVA. (**C**) Representative Western blot of iPSC-astrocyte lysates with or without TIC treatment for 24 h. Actin is shown as a loading control. (**D-G**) Western blot analysis of total and phosphorylated MAPK (p42/p44), STAT2, STAT3 and NFκB under basal conditions or after 24 h TIC treatment. Note that one outlier was removed (grey arrow in panel A), due to a z score of 3.47. Data represent up to 4 independent batches and up to 7 technical repeats using 5 controls and 3 *PSEN1* mutant lines (see Table S1). For data separated by iPSC line, see Fig S8. (**H-J**) qPCR analysis of *ISG15*, *OAS1* and *CXCL10*; genes involved in interferon response (associated with JAK-STAT2 signalling). Note that *CXCL10* was rarely detectable in untreated conditions (n.d.). Data represent up to 5 independent batches and up to 6 technical repeats from 6 control lines and 3 *PSEN1* lines, see Table S1. For data separated by iPSC line, see Fig S8. (**K**) High content imaging analysis of nuclear NFκB normalised to cytosolic NFκB and plotted as a fold-change relative to maximum signal. Data represent 2 control lines and 3 PSEN1 lines, with two experimental repeats (see Table S1). Individual data presented in Fig S8. Pairwise comparisons represent two tailed t-tests, where * = *p* < 0.05, ** = *p* < 0.01, *** = *p* < 0.001, and **** = *p* < 0.0001

To investigate cell signalling associated with STAT2, we first performed FACs analysis for cell surface levels of IFNAR2, a major interferon receptor associated with STAT2 signalling [55]. We observed a reduced level of cell surface IFNAR2 in one of the two *PSEN1* mutant lines tested, *PSEN1* int4del (Fig. 4A-B). This reduction remained significant after addition of TIC. Notably, *IFNAR2* expression is significantly upregulated in *PSEN1* mutant astrocytes compared to controls, both under basal conditions (*P* = 0.047) and after TIC treatment (*P* = 0.012) (Fig S3E).

Downstream of ligand (cytokine) binding, phosphorylated tyrosines at the intracellular domain of receptor tyrosine kinases initiate a phosphorylation cascade. Having found JAK-STAT2 as the most upregulated pathway in *PSEN1* mutant astrocytes, we performed Western blotting for phosphorylated, activated downstream signalling components including STAT2 and MAPK (p42/p44) (Fig. 4C-G).

We observed a significant reduction in phosphorylated MAPK in *PSEN1* mutant astrocytes under basal conditions (Fig. 4D). We also observed a reduced induction of STAT2 phosphorylation after TIC treatment in *PSEN1* cultures compared with healthy controls (Fig. 4E). We did not observe significant differences in STAT3 or NFκB signalling (Fig. 4F-G). To further validate these data, we quantified JAK-STAT2-associated gene expression by qPCR. We observed significant induction of *ISG15*, *OAS1* and *CXCL10* expression after TIC treatment in control and *PSEN1* mutant astrocytes (Fig. 4H-J). We also observed a significantly increased induction of *OAS1* in *PSEN1* astrocytes in response to TIC compared with controls (Fig. 4I), supporting data from transcriptomic analyses (e.g., *OAS1* in Fig. 3L). Finally, we used high content imaging to quantify nuclear NFκB in response to TIC over 24 h (Fig. 4K). We observed a slower recovery to baseline of nuclear NFκB signal in astrocytes harbouring *PSEN1* mutations, further supporting altered responses to inflammatory cues.

These data support altered phosphorylation of JAK-STAT2 signalling pathway components in response to inflammatory stimuli and further implicate deficits in regulated intramembrane proteolysis of cytokine receptors in *PSEN1* mutant astrocytes.

### Chemical manipulation of γ-secretase reinforces effects of *PSEN1* mutations on JAK-STAT2 signalling

To investigate the effect of γ-secretase activity on inflammatory responses, we treated astrocytes with TIC in combination with a γ-secretase inhibitor (GSI, DAPT) and a γ-secretase modulator (GSM, E2012). -secretase modulation has been shown to increase processivity [56, 57] as well as endoproteolytic activity at high concentrations [58], therein enhancing γ-secretase activity, which is in contrast to γ-secretase inhibition.

**Fig. 5:**
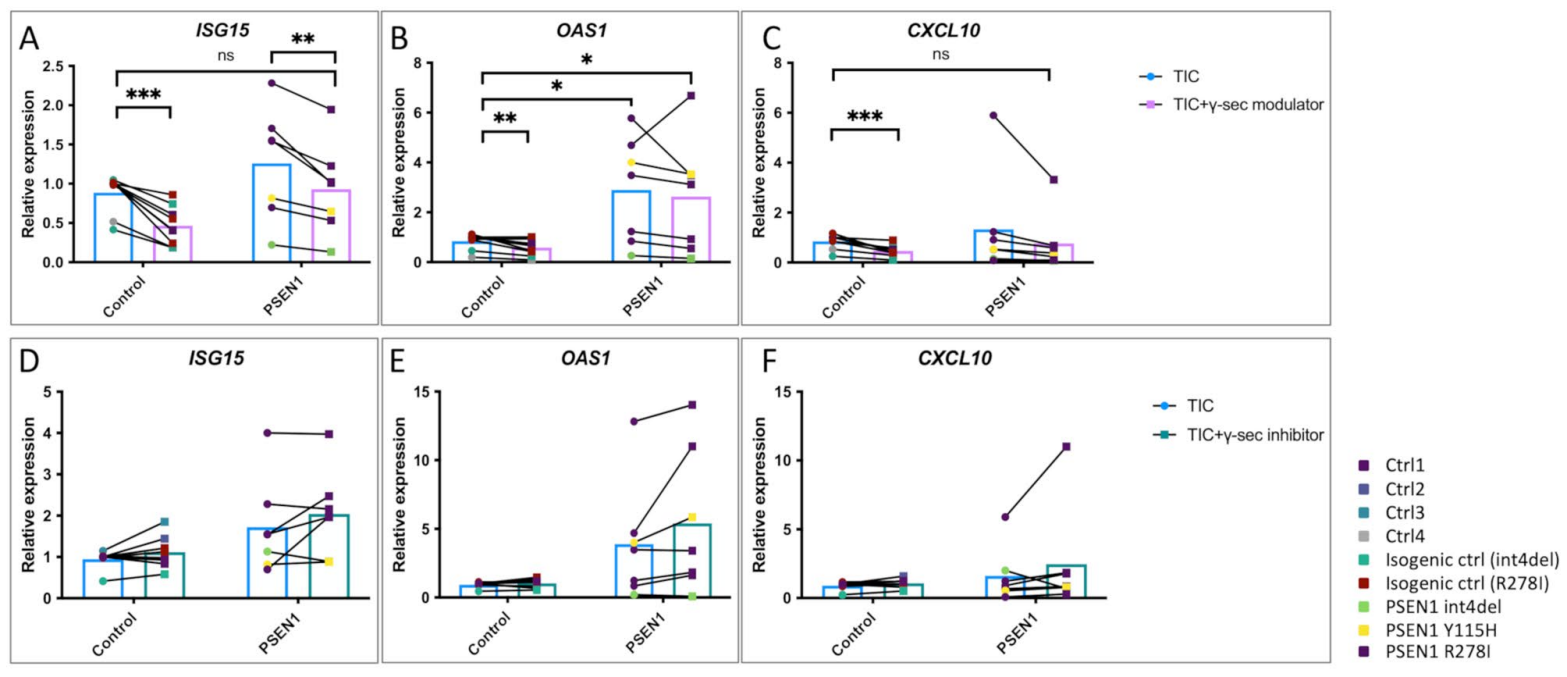
Chemical modulation of γ-secretase reduces interferon-responsive gene expression. (**A-C**) qPCR analysis of interferon responsive gene (*ISG15*, *OAS1* and *CXCL10*) expression after treatment of iPSC-astrocytes with TIC for 24 h in combination with the γ-secretase modulator (GSM) E2012. Data represent up to 3 independent batches and up to 4 technical repeats from 4 control lines and 3 *PSEN1* lines, see Table S1. (**D-E**) qPCR analysis of interferon responsive gene (*ISG15*, *OAS1* and *CXCL10*) expression after treatment of iPSC-astrocytes with TIC for 24 h in combination with the γ-secretase inhibitor (GSI) DAPT. Data represent up to 3 independent batches and up to 3 technical repeats from 5 control lines and 3 *PSEN1* lines, see Table S1. Pairwise comparisons represent paired, two tailed t-tests, where * = *p* < 0.05, ** = *p* < 0.01, *** = *p* < 0.001, and **** = *p* < 0.0001

Opposing trends were evident in GSM compared with GSI treated cultures. qPCR analyses demonstrated that healthy control astrocytes treated with GSM and TIC had a significantly lower induction of JAK-STAT2 readout genes (*ISG15*, *OAS1* and *CXCL10*) than cultures treated with TIC alone (Fig. 5A-C). Similar results were observed for *PSEN1* mutant astrocytes, with *ISG15* expression showing a significant decrease in TIC-GSM compared with TIC alone (Fig. 5A). In contrast, GSI and TIC treated cultures did not significantly differ from TIC treated astrocytes, although the trend was to stronger inflammatory responses with GSI plus TIC compared with TIC alone (Fig. 5D-F). Finally, it was interesting to note that GSM treated cultures displayed a higher degree of TNFR1 shedding compared to their untreated counterparts, although this was not the case in cultures with TIC (Fig S3C).

Together, these data support the finding that manipulating γ-secretase activity has a functional effect on inflammatory responses.

## Discussion

Using patient derived astrocytes, our data reveal that PSEN1 has a role in the glial response to inflammatory cues. The consequence of fAD-associated mutations in *PSEN1* is altered inflammatory cytokine responses and an enhanced basal inflammatory state in iPSC-derived astrocytes. Together, these data suggest that *PSEN1* mutations predispose to inflammation.

Specifically, fAD-associated mutations in *PSEN1* disrupt expression of genes associated with membrane proteolysis, evident via an upregulation of *PSEN2*, *PSENEN*, *ADAM10* and *BACE1*. The data support altered cleavage of membrane receptors, shown via reduced relative β-secretase cleavage of APP, reduced cell surface expression of IFNAR2 and increased sTNFR1 in the supernatants. This leads to a blunted cytokine response, evident via reduced STAT2 signalling and delayed nuclear NFκB dynamics. These changes converge to lead to an increased baseline inflammatory profile of the *PSEN1* mutation patient-derived astrocytes.

A predisposition to inflammation would thereby support a two-hit hypothesis in fAD; whereby we propose that *PSEN1* mutations not only impact APP processing but also the cellular response to inflammation, a hypothesis supported by recent work in microglia [31]. Risk factors associated with late onset AD (LOAD) have been shown to link inflammation and disease progression [59, 60], and therefore a two-hit hypothesis of fAD would link disease mechanisms in fAD and LOAD. Indeed, recent work has specifically linked JAK-STAT2 and interferon signalling in astrocytes with resilience to AD pathology [61] and a role for interferon type I signalling in the aggregation and spread of tau pathology [62], further reinforcing the findings herein and the potential overlap between pathomechanisms in fAD and LOAD.

Astrocytes have been centrally implicated in Alzheimer’s disease pathobiology previously, such as via the neurotoxic astrocyte phenotype [2, 9, 63, 64]. However, astrocytes are often thought to represent secondary responders to microglial inflammation. The literature supports an early role for astrocyte dysfunction in Alzheimer’s disease [7, 8]. For example, investigations into astrocyte-associated SNPs have linked the cell type with the earliest, amyloid phase of disease, whereas microglial-enriched genes are closely associated with putative downstream tau pathology [6]. The data presented here demonstrate that *PSEN1* mutant astrocytes may respond to disease processes in a cell autonomous manner; i.e., independently from microglia, given that microglia are absent in our cultures and that *PSEN1* mutant astrocytes show predisposition to inflammation in the absence of extrinsic stimuli. In support of this, in vivo and ex vivo animal studies have shown that reduced astrocyte function is associated with reduced Aβ clearance and increased plaque load [65, 66]. Additionally, recently identified biomarkers in pre-manifest fAD CSF reveal several candidates that are also dysregulated in our fAD patient-derived astrocyte cultures (e.g., SMOC2, STMN2 and SLIT/PDLIM family genes); providing clinical relevance to our model and supporting the presence of astrocyte dysfunction decades before clinical onset [67]. The proinflammatory phenotype of fAD astrocytes is supported by independent iPSC studies which show increased cytokine secretion and reduced neuronal support [68].

Based on our transcriptomic data, we posit that mutations in *PSEN1* broadly impact regulated membrane proteolysis. As a candidate pathway for detailed characterisation, we selected type I interferon signalling; including *OAS1* as a readout which has been identified as an Alzheimer’s disease risk gene [69]. Although interferon signalling has been previously implicated in astrocytes in a disease context [70], it is intriguing that we observe no expression of interferon ligands in these cultures, even after TIC stimulation. Furthermore, we propose that the effects of *PSEN1* mutations may extend to other cell types such as microglia. This is supported by findings demonstrating a role for γ-secretase in microglial state transitions [31] and studies implicating microglial interferon signalling in Down’s syndrome [71].

Our data support an anti-inflammatory role for γ-secretase activity, demonstrated by a decreased interferon response in astrocytes pretreated with γ-secretase modulators (which increase γ-secretase processivity). These findings are relevant to translational efforts, including ongoing γ-secretase modulator trials and the unsuccessful clinical trials of γ-secretase inhibitors, which worsened disease-associated phenotypes concomitant with increased infections rates [72]. Additionally, loss of function mutations in *PSEN1*, *PSENEN* and *NCSTN* all cause familial hidradenitis suppurativa, a chronic inflammatory skin condition [73, 74], further supporting a putative anti-inflammatory role of γ-secretase.

## Conclusion

In conclusion, we propose that fAD mutations in *PSEN1* disrupt membrane proteolysis in iPSC-derived astrocytes, impacting the inflammatory state of the cells. Disrupted cytokine signalling may therefore predispose to age-related inflammation in the brain, contributing to the onset and progression of Alzheimer’s disease. This aligns fAD with pathomechanisms of disease in LOAD and could inform rationalised therapeutic intervention.

## Electronic supplementary material

Below is the link to the electronic supplementary material.

Supplementary Table 1

Supplementary Table 2

**Supplementary Figure 1**: CRISPR-Cas9 genetic correction of the *PSEN1* R278I mutation in iPSCs. (**A**) Sanger sequencing confirms the correction of the point mutation (T > G). (**B**) Low coverage whole genome sequencing confirms stable karyotype of the isogenic control iPSC line. **Supplementary Figure 2**: Astrocyte model of familial Alzheimer’s disease. (**A**) Western blotting of iPSC-neuron, iPSC-astrocyte, iPSC-microglia and brain lysates for proteins associated with fAD (PSEN1, PSEN2 and APP). GAPDH and Actin serve as loading controls. Lower panel shows conditioned media from the three iPSC cultures, for amyloidogenic processed sAPP (sAPPβ) relative to total shed APP. 3 independent batches are shown from control iPSC lines. (**B**) Cytokine array using conditioned media (3 batches pooled) from control and *PSEN1* Y115H iPSC-astrocytes, either untreated or treated with TIC for 24 h. TNFα and IL1α are highlighted in red, as the array may be confounded by recombinant factors added via the TIC treatment. (**C**) Lactate dehydrogenase assay to analyse cell death in iPSC-astrocyte culture with or without TIC treatment. 3 independent batches are shown for each iPSC line. (**D-E**) Concentration of Aβ species normalised to RNA content for the cell pellets. (**F**) Glutamate uptake assays for control and *PSEN1* mutant astrocytes with or without TIC treatment, Data represent 3 experiments from 2 independent batches, with 5 control and 3 *PSEN1* patient-derived lines. **Supplementary Figure 3**: Evidence of altered shedding of TNF receptor in *PSEN1* mutant astrocytes and cultures treated with γ-secretase modulator. (**A**) ELISA quantification of sTNFR1 in conditioned media normalised to RNA content of the cell pellet. (**B**) Shows data from A separated by iPSC line. (**C**) Quantification of sTNFR1 in conditioned media of cultures treated with γ-secretase modulators/inhibitors and in combination with TIC. Normalised to GSI within each group. (**D-E**) Transcriptomic data for TNFR1 (coded by the *TNFRSF1A* gene) and *IFNAR2*. Data represents 3 experimental repeats with 5 controls and 3 *PSEN1* mutant astrocyte lines. **Supplementary Figure 4**: Differential gene expression of astrocytes (genotype and TIC treatment) investigated via transcriptomics. Volcano plots to represent differentially expressed genes in (**A**) control vs. *PSEN1* astrocytes, (**B**) Control untreated versus control TIC-treated astrocytes, (**C**) *PSEN1* untreated versus *PSEN1* TIC-treated astrocytes. (**D**) Geneset enrichment scoring to quantify and rank inflammatory profiles. Rows represent 14 studies that describe inflammatory gene signatures. Each astrocyte transcriptomic signature from this study (columns) is scored against inflammatory signatures from each dataset (red-to-blue heatmap, Table S2) and ranked based on this score (green). Higher inflammatory scores are shown to the left. TIC treated samples and PSEN1 mutant samples are shifted to the left, representing higher inflammatory state. **Supplementary Figure 5**: Evidence of altered cytokine to cytokine receptor interactions via GSEA of *PSEN1* mutant versus healthy control astrocytes under basal conditions (53, 54). This analysis was performed on the iDEP 2.01 platform (75), where red is upregulated expression and green are downregulated expression. **Supplementary Figure 6**: DoRothEA analysis of untreated versus TIC treated control astrocytes. (**A**) Major pathways upregulated by TIC treatment include NFκB, TNFα and JAK-STAT signalling. Transcription factors driving DEGs include *NFKB1*, *RELA* and *STAT2*. **Supplementary Figure 7**: Data from Fig 2 presented as individual iPSC lines. **Supplementary Figure 8**: Data from Fig 4 presented as individual iPSC lines. **Supplementary Figure 9**: Full blots for cropped images from Figs 2, 3 and 4

## Data Availability

All data from this study is available upon request and transcriptomic data has been deposited on gene expression omnibus (GEO) (GSE298491). All other data and tools are available upon reasonable request.
