# Supplementary material for: Mutations in PSEN1 predispose inflammation in an astrocyte model of familial Alzheimer’s disease through disrupted regulated intramembrane proteolysis
Source: Mol Neurodegener. 2025 Jun 20;20:73. doi: 10.1186/s13024-025-00864-7 (PMC12181884; doi:10.1186/s13024-025-00864-7)
Supplement: Supplementary file 3 — Supplementary Figure 1: CRISPR-Cas9 genetic correction of the PSEN1 R278I mutation in iPSCs. (A) Sanger sequencing confirms the correction of the point mutation (T > G). (B) Low coverage whole genome sequencing confirms stable karyotype of the isogenic control iPSC line. Supplementary Figure 2: Astrocyte model of familial Alzheimer’s disease. (A) Western blotting of iPSC-neuron, iPSC-astrocyte, iPSC-microglia and brain lysates for proteins associated with fAD (PSEN1, PSEN2 and APP). GAPDH and Actin serve as loading controls. Lower panel shows conditioned media from the three iPSC cultures, for amyloidogenic processed sAPP (sAPPβ) relative to total shed APP. 3 independent batches are shown from control iPSC lines. (B) Cytokine array using conditioned media (3 batches pooled) from control and PSEN1 Y115H iPSC-astrocytes, either untreated or treated with TIC for 24 h. TNFα and IL1α are highlighted in red, as the array may be confounded by recombinant factors added via the TIC treatment. (C) Lactate dehydrogenase assay to analyse cell death in iPSC-astrocyte culture with or without TIC treatment. 3 independent batches are shown for each iPSC line. (D-E) Concentration of Aβ species normalised to RNA content for the cell pellets. (F) Glutamate uptake assays for control and PSEN1 mutant astrocytes with or without TIC treatment, Data represent 3 experiments from 2 independent batches, with 5 control and 3 PSEN1 patient-derived lines. Supplementary Figure 3: Evidence of altered shedding of TNF receptor in PSEN1 mutant astrocytes and cultures treated with γ-secretase modulator. (A) ELISA quantification of sTNFR1 in conditioned media normalised to RNA content of the cell pellet. (B) Shows data from A separated by iPSC line. (C) Quantification of sTNFR1 in conditioned media of cultures treated with γ-secretase modulators/inhibitors and in combination with TIC. Normalised to GSI within each group. (D-E) Transcriptomic data for TNFR1 (coded by the TNFRSF1A gene) and IFNAR2. Data [file 13024_2025_864_MOESM3_ESM.pptx]

## Slide 1
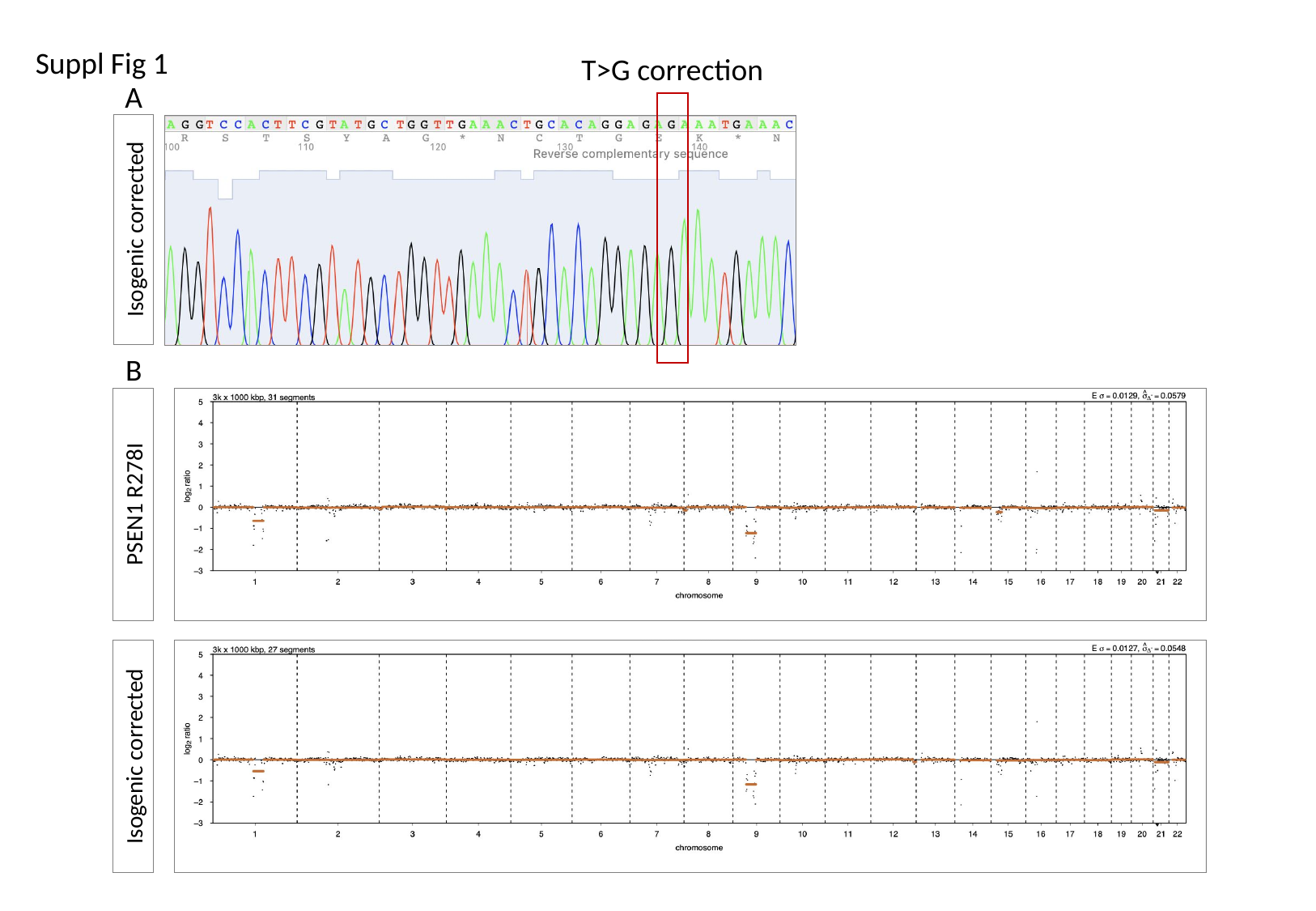

Suppl Fig 1
T>G correction
A
Isogenic corrected
B
PSEN1 R278I
Isogenic corrected

## Slide 2
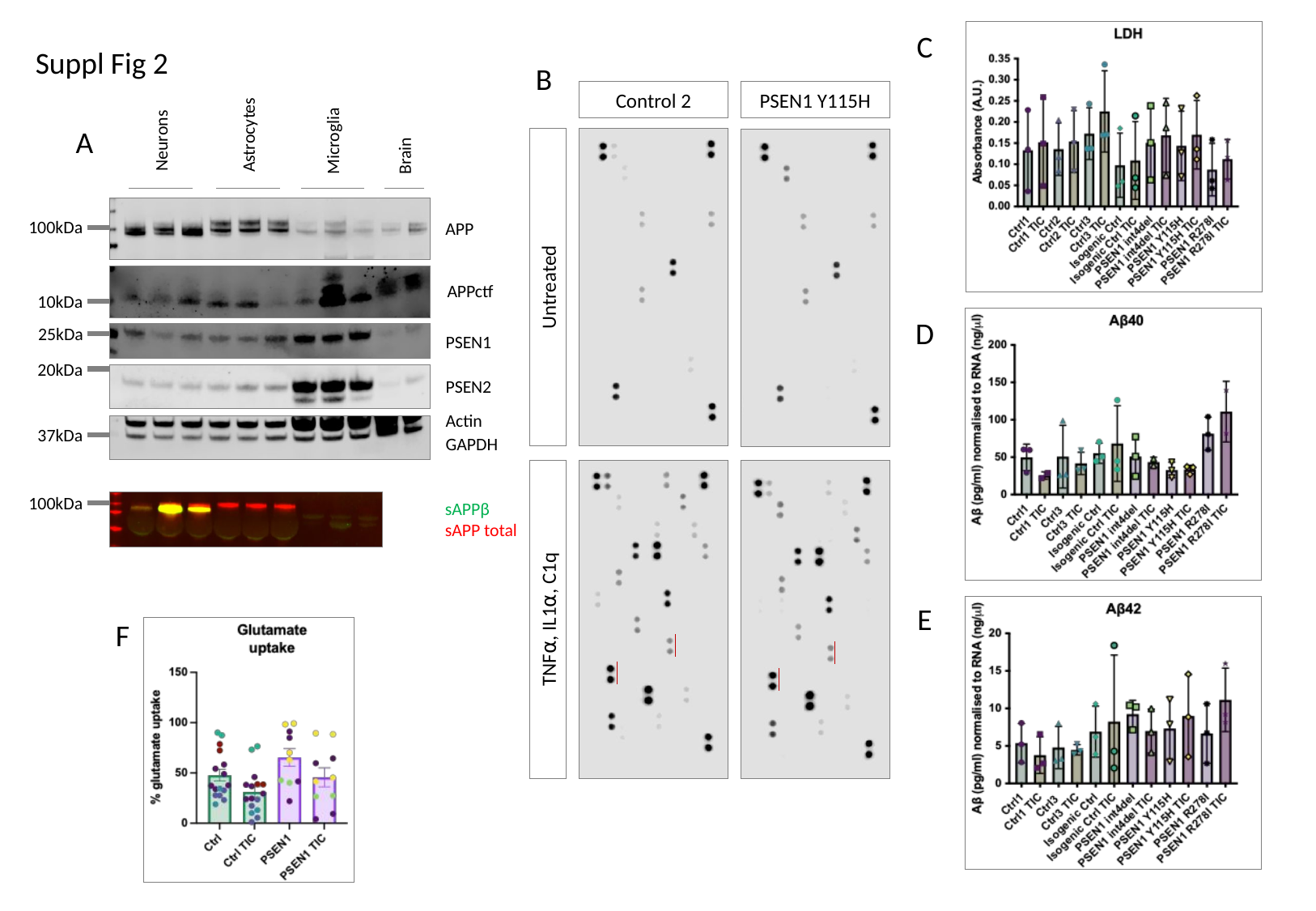

C
Suppl Fig 2
B
Control 2
PSEN1 Y115H
A
Astrocytes
Neurons
Microglia
Brain
100kDa
APP
Untreated
APPctf
10kDa
D
25kDa
PSEN1
20kDa
PSEN2
Actin
37kDa
GAPDH
100kDa
sAPPβ
sAPP total
E
TNF⍺, IL1⍺, C1q
F

## Slide 3
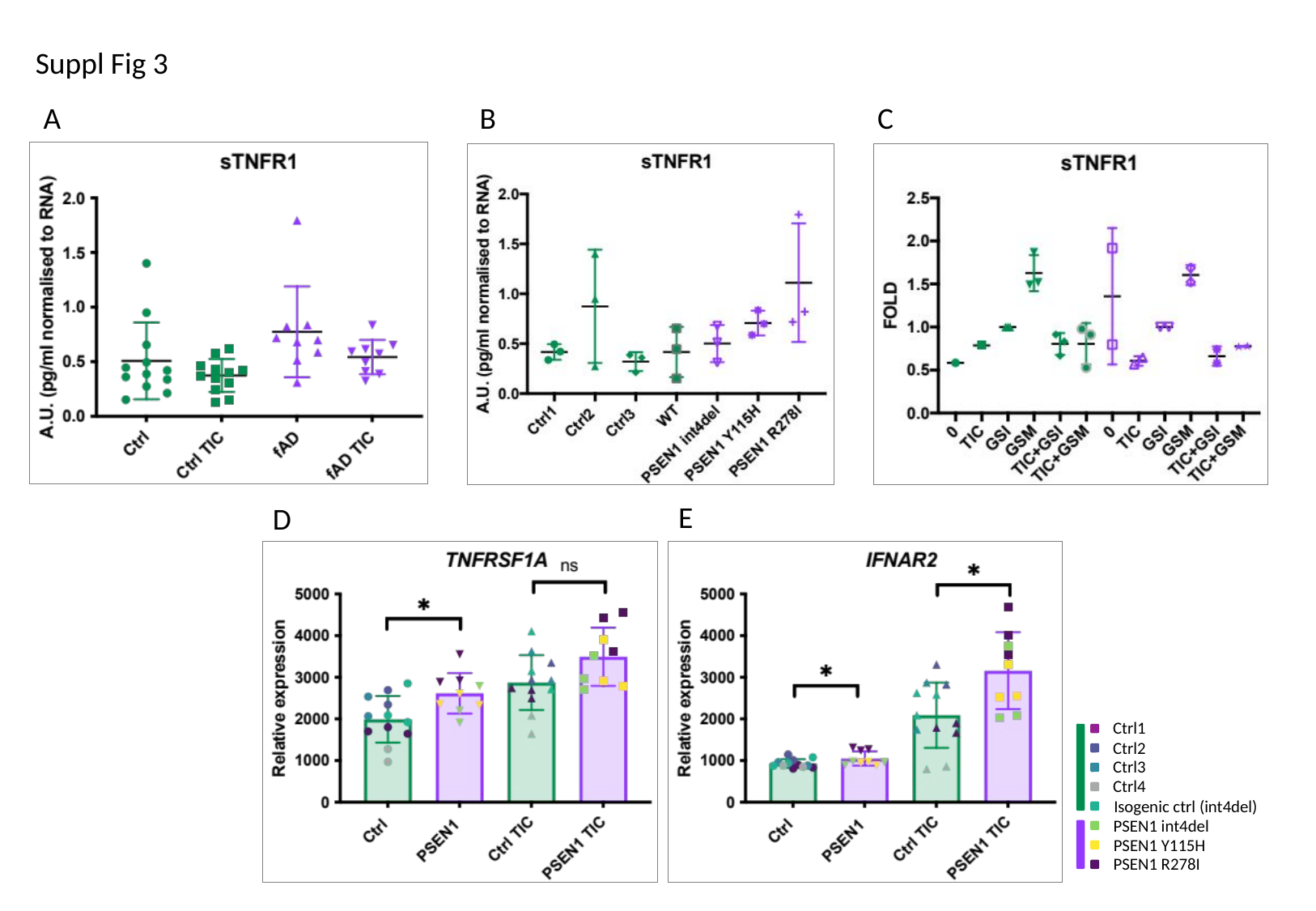

Suppl Fig 3
A
B
C
E
D
Ctrl1
Ctrl2
Ctrl3
Ctrl4
PSEN1 int4del
PSEN1 Y115H
PSEN1 R278I
Isogenic ctrl (int4del)

## Slide 4
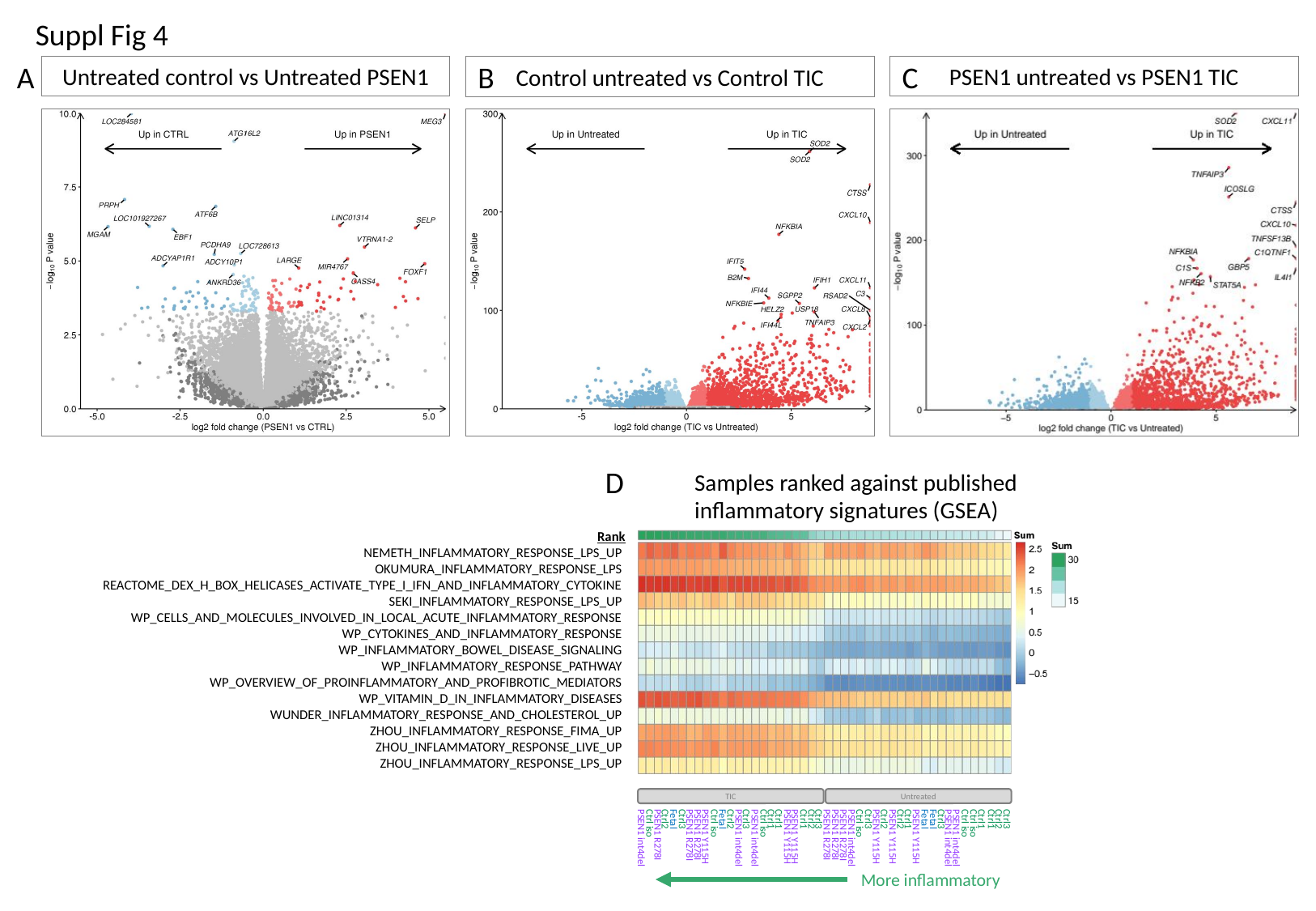

Suppl Fig 4
A
B
C
Untreated control vs Untreated PSEN1
PSEN1 untreated vs PSEN1 TIC
Control untreated vs Control TIC
D
Samples ranked against published inflammatory signatures (GSEA)
Rank
NEMETH_INFLAMMATORY_RESPONSE_LPS_UP
OKUMURA_INFLAMMATORY_RESPONSE_LPS
REACTOME_DEX_H_BOX_HELICASES_ACTIVATE_TYPE_I_IFN_AND_INFLAMMATORY_CYTOKINE
SEKI_INFLAMMATORY_RESPONSE_LPS_UP
WP_CELLS_AND_MOLECULES_INVOLVED_IN_LOCAL_ACUTE_INFLAMMATORY_RESPONSE
WP_CYTOKINES_AND_INFLAMMATORY_RESPONSE
WP_INFLAMMATORY_BOWEL_DISEASE_SIGNALING
WP_INFLAMMATORY_RESPONSE_PATHWAY
WP_OVERVIEW_OF_PROINFLAMMATORY_AND_PROFIBROTIC_MEDIATORS
WP_VITAMIN_D_IN_INFLAMMATORY_DISEASES
WUNDER_INFLAMMATORY_RESPONSE_AND_CHOLESTEROL_UP
ZHOU_INFLAMMATORY_RESPONSE_FIMA_UP
ZHOU_INFLAMMATORY_RESPONSE_LIVE_UP
ZHOU_INFLAMMATORY_RESPONSE_LPS_UP
TIC
Untreated
Ctrl2
Ctrl3
Ctrl2
Ctrl3
Ctrl1
Ctrl1
Ctrl1
Ctrl2
Ctrl3
Ctrl3
Ctrl2
Ctrl2
Ctrl1
Ctrl3
Ctrl1
Ctrl1
Ctrl2
Ctrl3
Fetal
Fetal
Fetal
Fetal
Ctrl iso
Ctrl iso
Ctrl iso
Ctrl iso
Ctrl iso
Ctrl iso
PSEN1 R278I
PSEN1 R278I
PSEN1 R278I
PSEN1 R278I
PSEN1 R278I
PSEN1 R278I
PSEN1 Y115H
PSEN1 Y115H
PSEN1 Y115H
PSEN1 Y115H
PSEN1 Y115H
PSEN1 Y115H
PSEN1 int4del
PSEN1 int4del
PSEN1 int4del
PSEN1 int4del
PSEN1 int4del
PSEN1 int4del
More inflammatory

## Slide 5
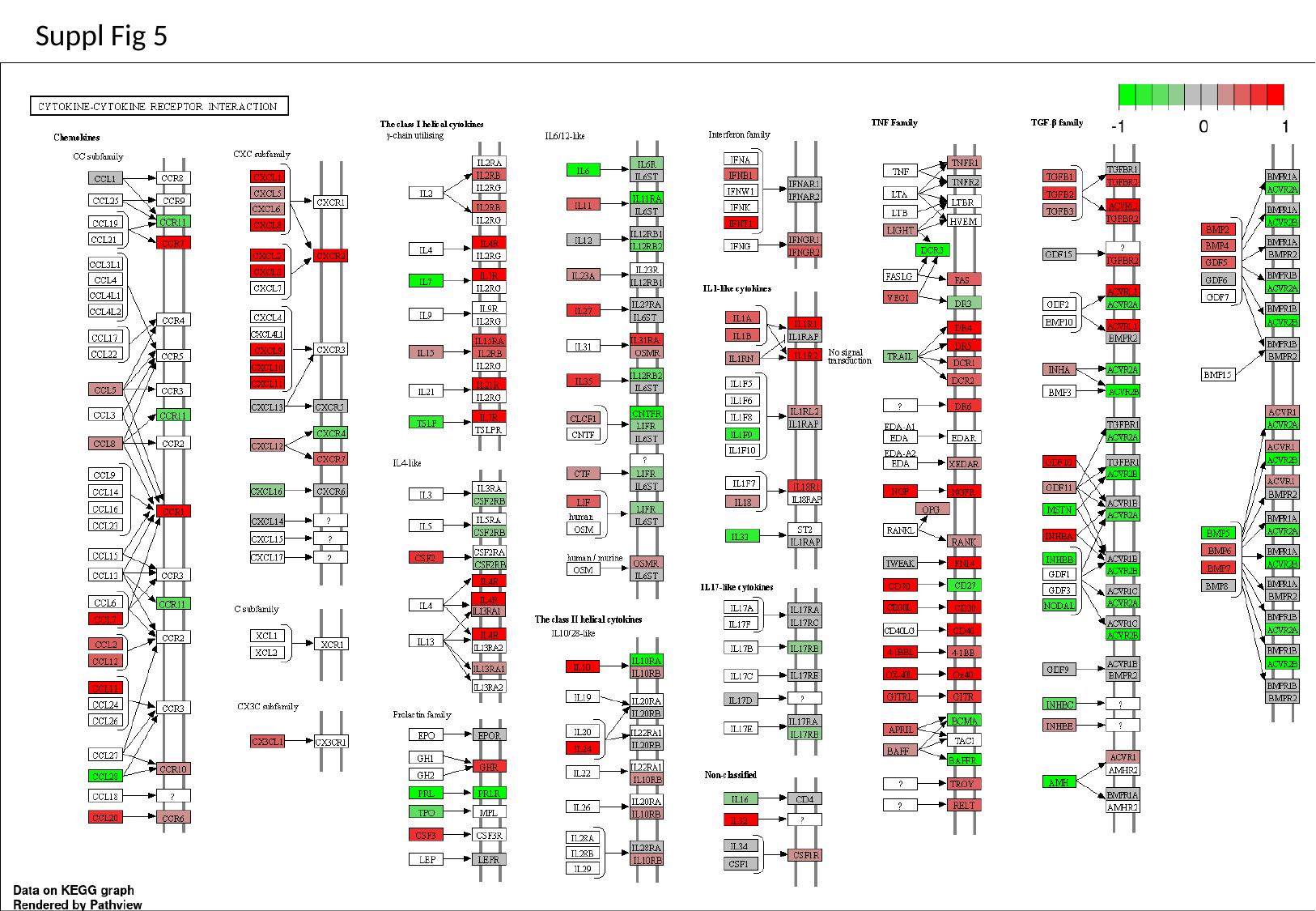

Suppl Fig 5

## Slide 6
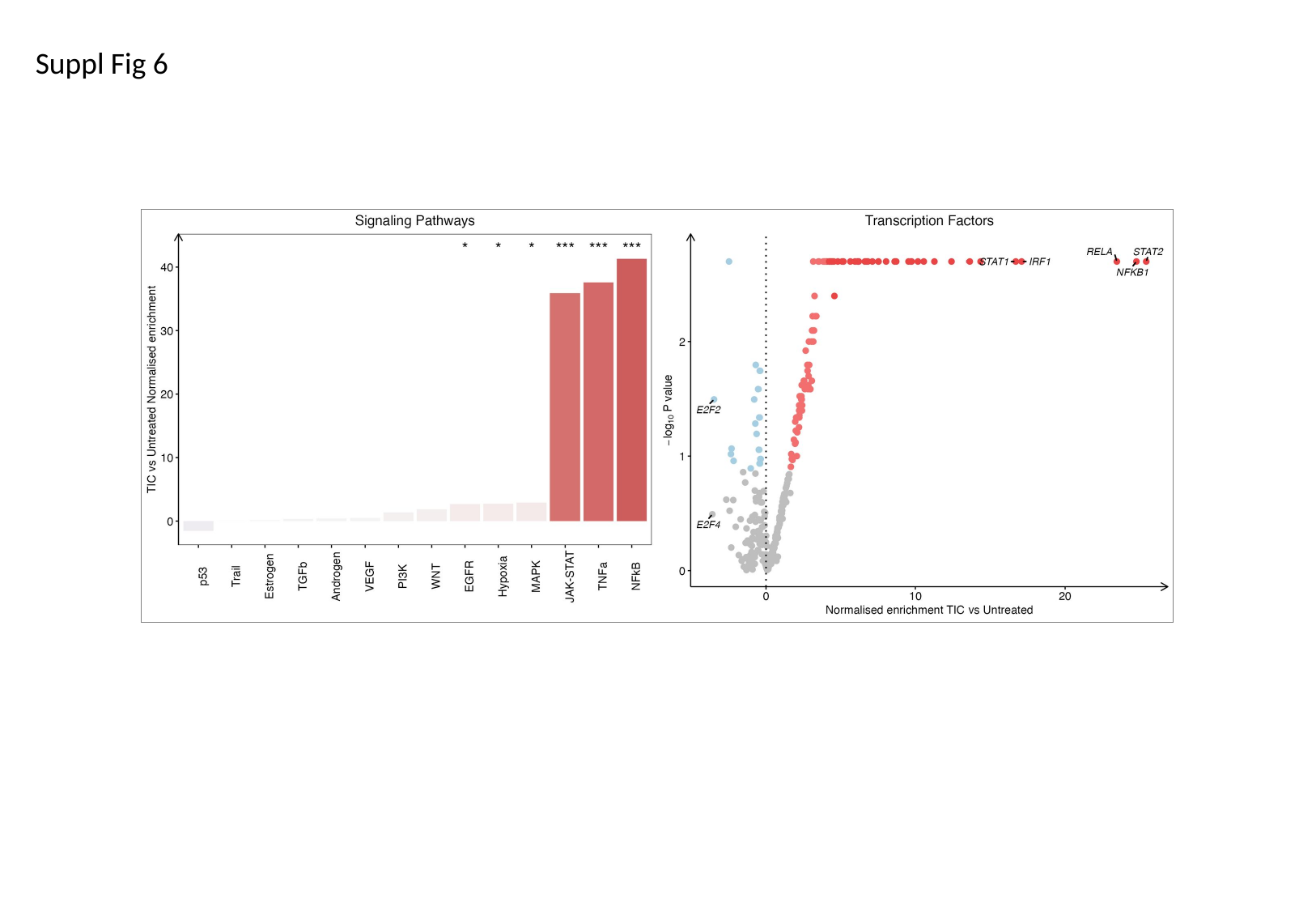

Suppl Fig 6

## Slide 7
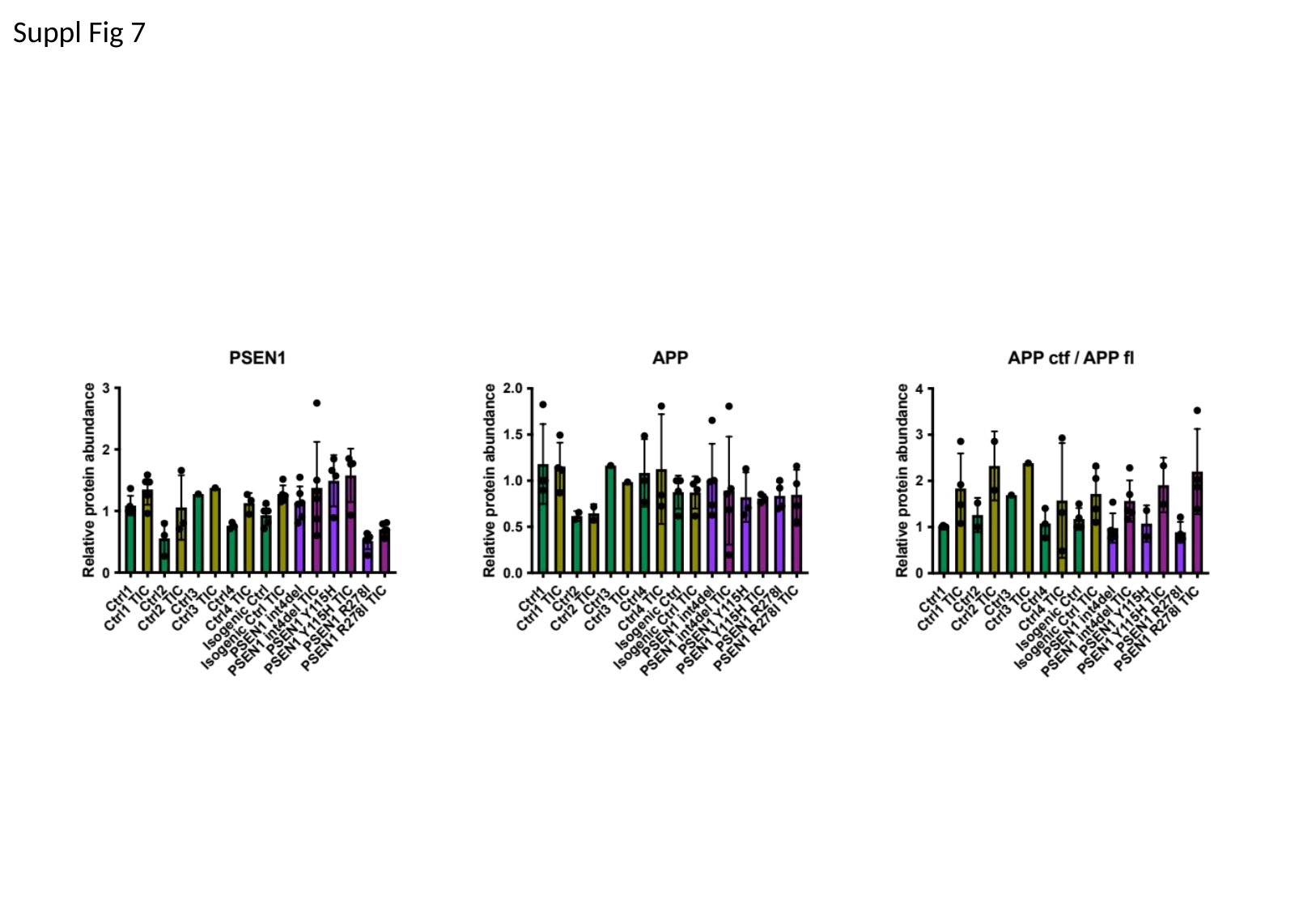

Suppl Fig 7

## Slide 8
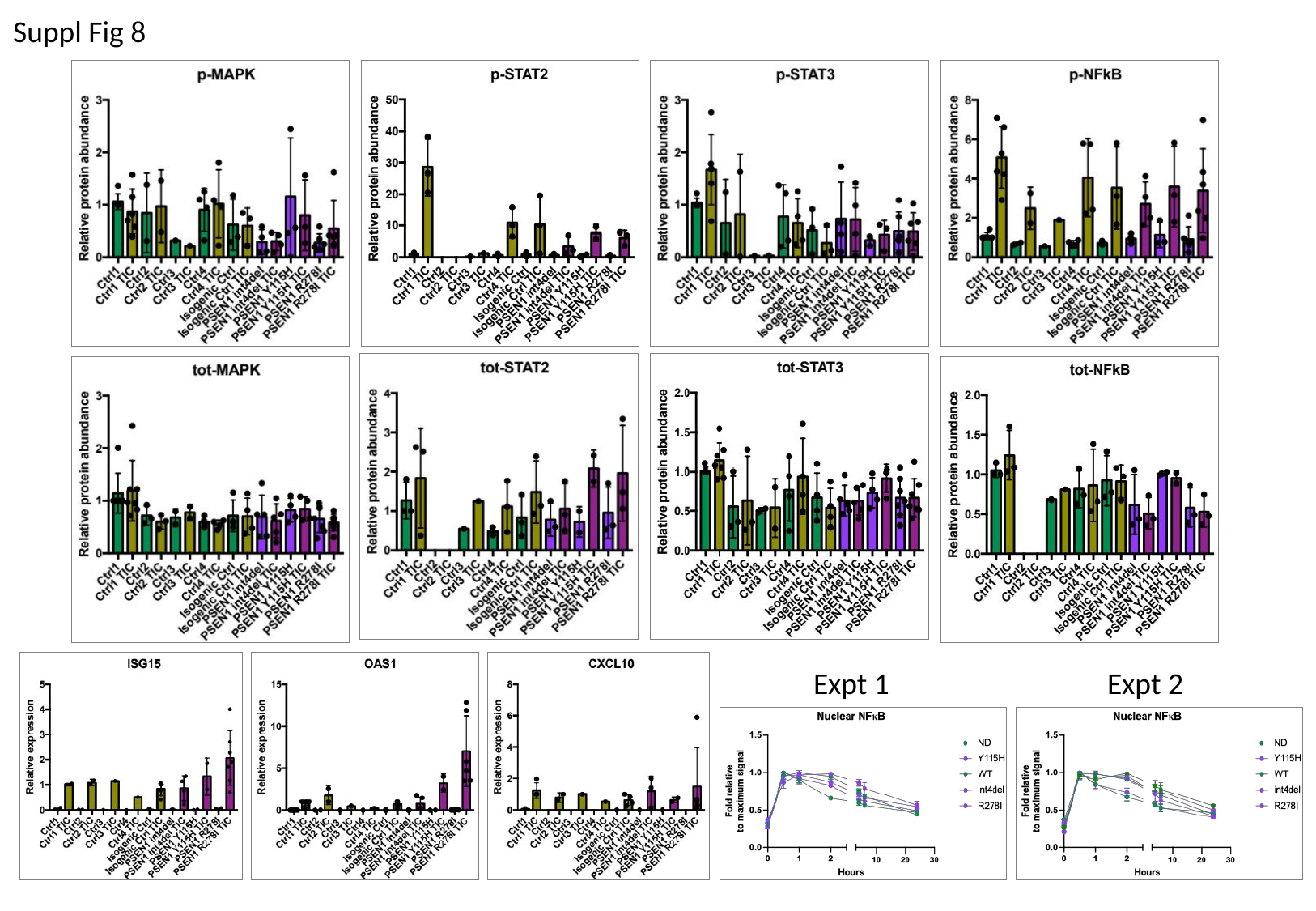

Suppl Fig 8
Expt 1
Expt 2

## Slide 9
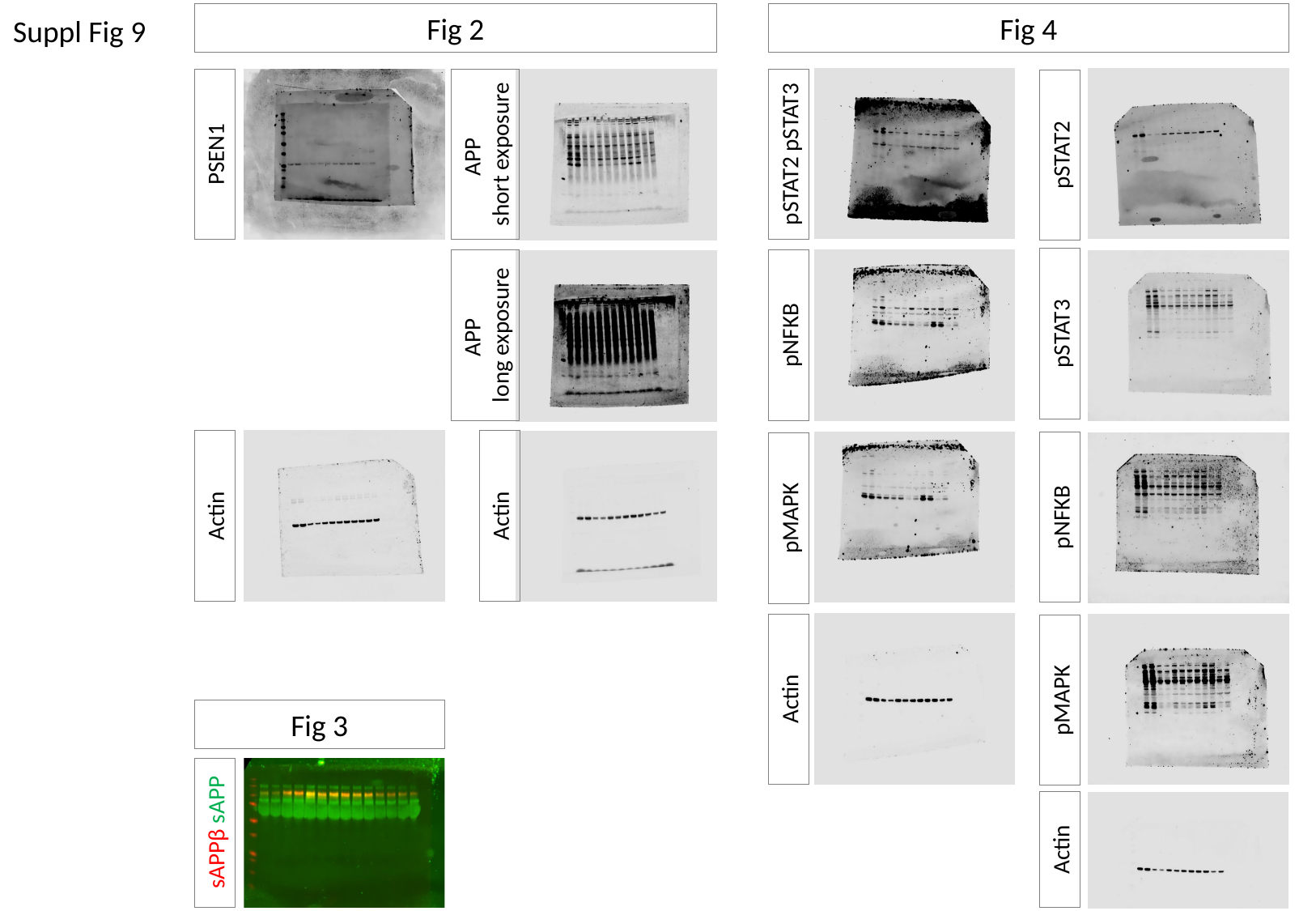

Fig 2
Fig 4
Suppl Fig 9
APP
short exposure
PSEN1
pSTAT2 pSTAT3
pSTAT2
APP
long exposure
pSTAT3
pNFKB
Actin
Actin
pNFKB
pMAPK
Actin
pMAPK
Fig 3
sAPPβ sAPP
Actin
